# Genome-wide identification and characterization of MADS-box family genes related to organ development and stress resistance in *Brassica rapa*

**DOI:** 10.1186/s12864-015-1349-z

**Published:** 2015-03-14

**Authors:** Gopal Saha, Jong-In Park, Hee-Jeong Jung, Nasar Uddin Ahmed, Md. Abdul Kayum, Mi-Young Chung, Yoonkang Hur, Yong-Gu Cho, Masao Watanabe, Ill-Sup Nou

**Affiliations:** Department of Horticulture, Sunchon National University, 413 Jungangno, Suncheon, Jeonnam 540-742 Republic of Korea; Department of Agricultural Education, Sunchon National University, 413 Jungangno, Suncheon, Jeonnam 540-742 Republic of Korea; Department of Biology, Chungnam National University, 96 Daehangno, Gung-dong, Yuseong-gu, Daejeon 305-764 Republic of Korea; Department of Crop Science, Chungbuk National University, 410 Seongbongro, Heungdokgu, Cheongju 361-763 Republic of Korea; Laboratory of Plant Reproductive Genetics, Graduate School of Life Sciences, Tohoku University, 2-1-1, Katahira, Aoba-ku, Sendai 980-8577 Japan

**Keywords:** MADS-box, Type I, Type II, MIKC^c^, Organ development, Abiotic stress, *Brassica rapa*

## Abstract

**Background:**

MADS-box transcription factors (TFs) are important in floral organ specification as well as several other aspects of plant growth and development. Studies on stress resistance-related functions of MADS-box genes are very limited and no such functional studies in *Brassica rapa* have been reported. To gain insight into this gene family and to elucidate their roles in organ development and stress resistance, we performed genome-wide identification, characterization and expression analysis of MADS-box genes in *B. rapa*.

**Results:**

Whole-genome survey of *B. rapa* revealed 167 MADS-box genes, which were categorized into type I (Mα, Mβ and Mγ) and type II (MIKC^c^ and MIKC*) based on phylogeny, protein motif structure and exon-intron organization. Expression analysis of 89 MIKC^c^ and 11 MIKC* genes was then carried out. In addition to those with floral and vegetative tissue expression, we identified MADS-box genes with constitutive expression patterns at different stages of flower development. More importantly, from a low temperature-treated whole-genome microarray data set, 19 *BrMADS* genes were found to show variable transcript abundance in two contrasting inbred lines of *B. rapa*. Among these, 13 *BrMADS* genes were further validated and their differential expression was monitored in response to cold stress in the same two lines via qPCR expression analysis. Additionally, the set of 19 *BrMADS* genes was analyzed under drought and salt stress, and 8 and 6 genes were found to be induced by drought and salt, respectively.

**Conclusion:**

The extensive annotation and transcriptome profiling reported in this study will be useful for understanding the involvement of MADS-box genes in stress resistance in addition to their growth and developmental functions, which ultimately provides the basis for functional characterization and exploitation of the candidate genes for genetic engineering of *B. rapa.*

**Electronic supplementary material:**

The online version of this article (doi:10.1186/s12864-015-1349-z) contains supplementary material, which is available to authorized users.

## Background

MADS-box genes play important roles in many aspects of plant development [1]. They are the major components in the well-known ‘ABC’ model that describes their roles in floral organ development [2]. MADS-box genes were identified initially as floral homeotic genes and are some of the most extensively studied transcription factors (TFs) involved in developmental control [3-5]. MADS-box proteins are characterized by the presence in the N-terminal region of a conserved MADS-box DNA-binding domain of approximately 58–60 amino acids that binds to so-called *CArG* boxes (CC[A/T]_6_GG) [6].

Plant MADS-box genes have been subdivided into two main groups *viz.* M-type*,* also designated as type I, and MIKC, also known as type II [7]. The M-type MADS-box genes are grouped into Mα, Mβ and Mγ based on phylogenetic relationships within their MADS-box regions [4]. The MIKC genes are characterized by the presence of keratin-like (K) domain and are classified as either MIKC^c^ or MIKC*-type [8]. The MIKC^c^ genes are further partitioned into 14 clades based on phylogeny [9].

MIKC-type proteins generally contain four common domains. In addition to the MADS (M) domain, MIKC proteins contain intervening (I), K and C-terminal (C) domains [10,11]. The I domain is relatively less conserved, and contributes to the DNA binding specificity and dimerization of these proteins [12]. The K domain is characterized by a coiled-coil structure that mainly functions in the dimerization of MADS-box proteins. The K domain, which is present in MIKC MADS-box proteins but absent from M-type proteins, is more highly conserved than the I domain [4,13], and the MIKC* group has longer I domains and less conserved K domains than the MIKC^c^ group [8]. The C domain, which is the least conserved, plays important roles in transcriptional activation and the formation of multimeric MADS-box protein complexes [14].

The most remarkable feature of the MADS-box gene family is the divergent functions of its members in different aspects of plant growth and development, such as flowering time control, meristem identity, floral organ identity, formation of the dehiscence zone, fruit ripening, embryo development and the development of vegetative organs such as roots and leaves [7,15-17]. Previous reports revealed the role of MIKC^c^ in reproductive organ development of higher plants, and this has been the well-characterized group of MADS-box proteins in plants. To date, MIKC^c^ genes have been found to play fundamental roles in flowering time (*SOC1* (*SUPPRESSOR OF OVERESPRESSION OF CONSTANS1*), *FLC1* (*FLOWERING LOCUS C*), *AGL24* (*AGAMOUS-LIKE GENE 24*), *MAF1/FLM* (*MADS AFFECTING FLOWERING*) and *SVP* (*SHORT VEGETATIVE PHASE*); [18]); floral meristem identity (*AP1* (*APETALA 1*), *FUL* (*FRUITFUL*) and *CAL* (*CAULIFLOWER*); [19]); the formation of floral organs (*AP1*, *SEP1-3* (*SEPALLATA 1–3*), *AP3* (*APETALA 3*), *PI* (*PISTILLATA*) and *AG* (*AGAMOUS*); [20]); fruit ripening (*SHP1, SHP2* (*SHATTERPROOF 1–2*) and *FUL*; [21,22]) and seed pigmentation and embryo development (*TT16* (*TRANSPARENT TESTA16*); [23]).

The biological functions of MIKC^c^ genes in flower organogenesis can be grouped into five classes, *A, B, C, D* and *E*, which are required in different combinations to specify the identity of sepals (*A + E*), petals (*A + B + E*), stamens (*B + C + E*), carpels (*C + E*) and ovules (*D + E*) [20,24,25]. Expression of MIKC^c^ genes has also been detected outside reproductive organs, e.g., of genes belonging to the *AGL12* and *AGL17* subfamilies [1,26]. This expression suggested a role for those genes in vegetative development, which was later demonstrated for some of them in root development. Nevertheless, *AGL12* and *AGL17* have been proposed to play roles as flowering promoters [27]. By contrast, M-type (type I) MADS-box genes in Arabidopsis appear to function exclusively during female gametophyte and seed development [28].

The genus *Brassica* includes a number of important crops that provide oil, vegetables, condiments, dietary fiber, and vitamin C [29]. Among *Brassica* species, *Brassica rapa* comprises several subspecies, including Chinese cabbage (*B. rapa* ssp. *pekinensis*), non-heading Chinese cabbage (*B. rapa* ssp. *chinensis*) and turnip (*B. rapa* ssp. *rapifera*). Chinese cabbage is one of the most important vegetables in Asia. In addition, *B. rapa* is used as the model species representing the *Brassica* ‘A’ genome and, therefore, was selected for genome sequencing [30,31]. This species has already proven a useful model for studying polyploidy, in part because it has a relatively small genome [approximately 529 megabase pairs (Mbp)] compared to other *Brassica* species. Comparative genomic analysis confirmed that *B. rapa* underwent genome triplication since its divergence from Arabidopsis [32]. MADS-box family genes have been thoroughly studied in its close relative Arabidopsis, but have not been characterized in the relatively large and complex genome of *B. rapa*. Over the course of evolution, the number of genes in this family steadily increased as the reproductive system became more complex; concomitant with this expansion of the lineage, MADS-box genes have been found to perform more diversified functions [33]. In addition to growth and development-related functions, some stress-responsive MADS-box genes have also been reported in wheat and rice [34,35]. As an important vegetable crop world-wide, *Brassica* species are subject to a variety of abiotic stresses. Identification of stress-resistance-related MADS-box genes in *Brassica* could be highly useful.

The recent sequencing of the *Brassica rapa* ssp. *pekinensis* genome [36] offers the possibility of genome-wide analysis of MADS-box genes. In this study, we analyzed the genomic localization, protein motif structure, phylogenetic relationships, and gene structure of all candidate MADS-box genes in *B. rapa*. We carried out extensive expression profiling for specific MIKC^c^ subfamilies in vegetative and reproductive organs, as well as during flower developmental stages. Additionally, we investigated a considerable number of MADS-box genes, selected from whole-genome, low temperature-treated microarray data in the cold-tolerant and -susceptible inbred lines of *B. rapa*, Chiifu and Kenshin, respectively.

## Results

### Identification and sequence analysis of MADS-box genes in *B. rapa*

A set of 167 candidate MADS-box genes from the *B. rapa* genome was recovered using key word ‘MADS-box’ to search Swissprot annotations at the *Brassica* database (BRAD) (http://brassicadb.org/brad/) [37]. This number of candidates *B. rapa* (167) is higher than the number of MADS-box genes in Arabidopsis, rice, soybean, maize and sorghum (Additional file 1: Table S1) [4,35,38,39]. A domain search using EMBL (http://smart.embl.de/smart/set_mode.cgi?GENOMIC=1) with the corresponding *B. rapa* candidate protein sequences confirmed 162 of them to contain a ‘MADS’ domain, whereas the other 5 did not. The five candidates (BrMADS85, 87, 89, 119 and 127) that lacked a ‘MADS’ domain shared considerable sequence similarity with MADS-box proteins of other crop species that also lack ‘MADS’ domains and are considered to be MADS-box proteins (4 published and 1 unpublished MADS-box genes; Additional file 1: Table S2). We classified all 167 putative *B. rapa* MADS-box proteins into five classes (i. e., MIKC^c^ and MIKC* of type II and Mα, Mβ and Mγ of type I) in accord with the previously reported classification of the MADS-box family members in flowering plants [4]. We designated the 167 annotated MADS-box genes of *B. rapa* as *BrMADS* followed by Arabic numbers 1–167, consecutively following the five classes (MIKC^c^, MIKC*, Mα, Mβ and Mγ). Subsequent sequence analysis of the 167 genes showed open reading frame (ORFs) ranging from 180 to 2379 bp and predicted protein lengths from 59 to 792 amino acid (data not shown). Sequence analysis also revealed that *B. rapa* MIKC (type II) MADS-box genes usually contained multiple introns, with a maximum of 15 introns; the exceptions were *BrMADS84, BrMADS86* and *BrMADS88*, which did not have any introns. Almost all of the M-type (type I) genes lacked introns or had only a single intron; however, M-type MADS-box genes *BrMADS109* and *BrMADS119* had 3 and 2 introns respectively (Table 1 and Additional file 2: Figure S2). These features are consistent with those of MADS-box genes in other flowering plants such as Arabidopsis, rice, grapevine, and soybean [4,13,35,38].

**Table 1:** ***In silico*** **analysis of 167 MADS-box genes identified in** ***B. rapa*** **with their closest** ***Arabidopsis*** **homologs and sequence characteristics (aa, amino acids; Kda, Kilo dalton)**

**Sl no.**	**Gene name**	**Gene locus**	**Chr. no.**	**Closest arabidopsis homolog**	**Protein**		**No. of introns**	**Group**
					**Length (aa)**	**Mol.wt. (Kda)**		
1	*BrMADS1*	Bra040348	A08	*AGL18*	293	32.69	5	MIKC^c^
2	*BrMADS2*	Bra014628	A04	*AGL18*	250	28.02	7	MIKC^c^
3	*BrMADS3*	Bra007324	A09	*AGL18*	255	28.59	7	MIKC^c^
4	*BrMADS4*	Bra019018	A06	*AGL18*	200	22.90	6	MIKC^c^
5	*BrMADS5*	Bra008802	A10	*AGL15*	264	30.13	7	MIKC^c^
6	*BrMADS6*	Bra006214	A03	*AGL15*	264	30.00	7	MIKC^c^
7	*BrMADS7*	Bra031888	A02	*AGL69*	178	19.84	5	MIKC^c^
8	*BrMADS8*	Bra024350	A06	*AGL27/FLM*	196	22.43	6	MIKC^c^
9	*BrMADS9*	Bra031886	A02	*AGL69*	250	28.14	6	MIKC^c^
10	*BrMADS10*	Bra024351	A06	*AGL27/FLM*	200	22.75	6	MIKC^c^
11	*BrMADS11*	Bra031884	A02	*AGL27/FLM*	199	22.80	6	MIKC^c^
12	*BrMADS12*	Bra028599	A02	*AGL25/FLC*	196	21.93	6	MIKC^c^
13	*BrMADS13*	Bra009055	A10	*AGL25/FLC*	206	22.94	6	MIKC^c^
14	*BrMADS14*	Bra006051	A03	*AGL25/FLC*	197	21.64	6	MIKC^c^
15	*BrMADS15*	Bra022771	A03	*AGL25/FLC*	143	16.04	4	MIKC^c^
16	*BrMADS16*	Bra039921	A09	*AGL17*	227	26.38	6	MIKC^c^
17	*BrMADS17*	Bra030222	A04	*AGL17*	227	26.18	6	MIKC^c^
18	*BrMADS18*	Bra011797	A01	*AGL21*	228	33.78	6	MIKC^c^
19	*BrMADS19*	Bra010623	A08	*AGL21*	214	24.65	5	MIKC^c^
20	*BrMADS20*	Bra017638	A03	*AGL16*	240	27.51	6	MIKC^c^
21	*BrMADS21*	Bra011509	A01	*AGL16*	290	40.19	6	MIKC^c^
22	*BrMADS22*	Bra038511	A09	*AGL22/SVP*	241	27.31	8	MIKC^c^
23	*BrMADS23*	Bra030228	A04	*AGL22/SVP*	236	26.78	7	MIKC^c^
24	*BrMADS24*	Bra019221	A03	*AGL24*	216	24.55	6	MIKC^c^
25	*BrMADS25*	Bra013812	A01	*AGL24*	792	88.94	15	MIKC^c^
26	*BrMADS26*	Bra029365	A02	*AGL32/TT16*	242	28.44	5	MIKC^c^
27	*BrMADS27*	Bra026507	A01	*AGL32/TT16*	300	36.72	7	MIKC^c^
28	*BrMADS28*	Bra013028	A03	*AGL32/TT16*	240	28.11	6	MIKC^c^
29	*BrMADS29*	Bra020093	A02	*PISTILLATA*	203	23.38	5	MIKC^c^
30	*BrMADS30*	Bra006549	A03	*PISTILLATA*	208	24.05	4	MIKC^c^
31	*BrMADS31*	Bra002285	A10	*PISTILLATA*	146	16.62	3	MIKC^c^
32	*BrMADS32*	Bra014822	A04	*APETALA3*	224	26.39	6	MIKC^c^
33	*BrMADS33*	Bra007067	A09	*APETALA3*	232	27.28	6	MIKC^c^
34	*BrMADS34*	Bra007972	A02	*AGL12*	211	23.99	6	MIKC^c^
35	*BrMADS35*	Bra003919	A07	*AGL12*	212	24.00	6	MIKC^c^
36	*BrMADS36*	Bra039324	A04	*AGL20/SOC1*	213	24.35	6	MIKC^c^
37	*BrMADS37*	Bra000393	A03	*AGL20/SOC1*	213	24.35	6	MIKC^c^
38	*BrMADS38*	Bra004928	A05	*AGL20/SOC1*	213	24.40	6	MIKC^c^
39	*BrMADS39*	Bra029424	A09	*AGL14*	173	19.78	4	MIKC^c^
40	*BrMADS40*	Bra020826	A08	*AGL19*	146	16.16	2	MIKC^c^
41	*BrMADS41*	Bra013662	A01	*AGL19*	718	81.80	10	MIKC^c^
42	*BrMADS42*	Bra019343	A03	*AGL19*	219	25.07	6	MIKC^c^
43	*BrMADS43*	Bra035907	A09	*AGL42*	272	31.73	9	MIKC^c^
44	*BrMADS44*	Bra029281	A02	*AGL42*	209	24.74	6	MIKC^c^
45	*BrMADS45*	Bra029314	A02	*AGL72*	187	21.99	3	MIKC^c^
46	*BrMADS46*	Bra013891	A01	*AGL72*	189	21.94	3	MIKC^c^
47	*BrMADS47*	Bra010465	A08	*AGL72*	187	21.31	2	MIKC^c^
48	*BrMADS48*	Bra012957	A03	*AGL72*	211	24.14	5	MIKC^c^
49	*BrMADS49*	Bra029155	A03	*AGL72*	209	23.90	6	MIKC^c^
50	*BrMADS50*	Bra028282	A01	*AGL72*	202	23.37	6	MIKC^c^
51	*BrMADS51*	Bra029154	A03	*AGL71*	219	25.46	6	MIKC^c^
52	*BrMADS52*	Bra028283	A01	*AGL71*	199	23.05	5	MIKC^c^
53	*BrMADS53*	Bra037895	A09	*AGL11*	230	26.27	6	MIKC^c^
54	*BrMADS54*	Bra000696	A03	*AGL11*	231	26.38	6	MIKC^c^
55	*BrMADS55*	Bra013364	A01	*AGAMOUS*	252	28.78	6	MIKC^c^
56	*BrMADS56*	Bra012564	A03	*AGAMOUS*	251	28.77	6	MIKC^c^
57	*BrMADS57*	Bra014552	A04	*AGL1/SHP1*	248	28.39	6	MIKC^c^
58	*BrMADS58*	Bra003356	A07	*AGL1/SHP1*	273	31.26	6	MIKC^c^
59	*BrMADS59*	Bra007419	A09	*AGL1/SHP1*	245	27.76	6	MIKC^c^
60	*BrMADS60*	Bra004716	A05	*AGL5/SHP2*	244	28.01	5	MIKC^c^
61	*BrMADS61*	Bra038326	A02	*AGL7/AP1*	256	30.12	7	MIKC^c^
62	*BrMADS62*	Bra004361	A07	*AGL7/AP1*	189	22.51	5	MIKC^c^
63	*BrMADS63*	Bra004007	A07	*AGL7/AP1*	271	31.67	8	MIKC^c^
64	*BrMADS64*	Bra035952	A09	*AGL8/FUL*	241	27.50	7	MIKC^c^
65	*BrMADS65*	Bra029347	A02	*AGL8/FUL*	240	27.34	7	MIKC^c^
66	*BrMADS66*	Bra012997	A03	*AGL8/FUL*	241	27.45	7	MIKC^c^
67	*BrMADS67*	Bra036201	A09	*AGL79*	248	27.97	7	MIKC^c^
68	*BrMADS68*	Bra025411	A06	*AGL79*	176	20.25	5	MIKC^c^
69	*BrMADS69*	Bra020742	A02	*AGL79*	577	64.08	9	MIKC^c^
70	*BrMADS70*	Bra011021	A08	*AGL10/CAL*	254	29.88	6	MIKC^c^
71	*BrMADS71*	Bra014454	A04	*AGL13*	230	26.21	6	MIKC^c^
72	*BrMADS72*	Bra004927	A05	*AGL6*	242	27.60	7	MIKC^c^
73	*BrMADS73*	Bra000392	A03	*AGL6*	257	29.47	7	MIKC^c^
74	*BrMADS74*	Bra021470	A01	*AGL4/SEP2*	252	28.77	6	MIKC^c^
75	*BrMADS75*	Bra039170	A05	*AGL4SEP2*	250	28.57	6	MIKC^c^
76	*BrMADS76*	Bra010955	A08	*AGL9/SEP3*	244	28.21	7	MIKC^c^
77	*BrMADS77*	Bra032814	A09	*AGL9/SEP3*	253	29.32	7	MIKC^c^
78	*BrMADS78*	Bra026543	A02	*AGL3/SEP4*	269	30.63	7	MIKC^c^
79	*BrMADS79*	Bra017376	A09	*AGL3/SEP4*	243	27.76	7	MIKC^c^
80	*BrMADS80*	Bra025126	A06	*AGL3/SEP4*	257	29.41	7	MIKC^c^
81	*BrMADS81*	Bra030032	A07	*AGL9/SEP3*	252	29.25	7	MIKC^c^
82	*BrMADS82*	Bra008674	A10	*AGL2/SEP1*	252	28.78	6	MIKC^c^
83	*BrMADS83*	Bra006322	A03	*AGL2/SEP1*	250	28.55	6	MIKC^c^
84	*BrMADS84*	Bra003278	A07	*AGL18*	61	6.91	0	MIKC^c^
85	*BrMADS85*	Bra003279	A07	*AGL18*	197	22.06	6	MIKC^c^
86	*BrMADS86*	Bra005545	A05	*AGL18*	59	6.90	0	MIKC^c^
87	*BrMADS87*	Bra029494	A09	*AGL15*	118	13.66	3	MIKC^c^
88	*BrMADS88*	Bra016128	A07	*AGL12*	62	7.04	0	MIKC^c^
89	*BrMADS89*	Bra019163	A03	*AGL72*	172	19.64	4	MIKC^c^
90	*BrMADS90*	Bra011763	A01	*AGL67*	175	20.52	5	MIKC^*^
91	*BrMADS91*	Bra015645	A07	*AGL67*	209	24.60	7	MIKC^*^
92	*BrMADS92*	Bra012308	A07	*AGL104*	335	38.15	9	MIKC^*^
93	*BrMADS93*	Bra016386	A08	*AGL104*	311	35.23	7	MIKC^*^
94	*BrMADS94*	Bra015643	A07	*AGL66*	329	37.59	8	MIKC^*^
95	*BrMADS95*	Bra025685	A06	*AGL65*	379	43.18	9	MIKC^*^
96	*BrMADS96*	Bra016544	A08	*AGL65*	306	35.12	5	MIKC^*^
97	*BrMADS97*	Bra031049	A09	*AGL65*	382	43.92	9	MIKC^*^
98	*BrMADS98*	Bra024792	A06	*AGL30*	377	42.65	10	MIKC^*^
99	*BrMADS99*	Bra017404	A09	*AGL30*	379	42.78	8	MIKC^*^
100	*BrMADS100*	Bra004393	A07	*AGL94*	349	40.09	7	MIKC^*^
101	*BrMADS101*	Bra040149	A01	*AGL57*	174	19.90	0	Mα
102	*BrMADS102*	Bra037759	A09	*AGL58*	190	21.24	0	Mα
103	*BrMADS103*	Bra031945	A02	*AGL57*	193	22.17	0	Mα
104	*BrMADS104*	Bra032347	A09	*AGL64*	186	20.77	0	Mα
105	*BrMADS105*	Bra038225	A01	*AGL28*	261	30.31	1	Mα
106	*BrMADS106*	Bra022434	A05	*AGL62*	283	32.41	1	Mα
107	*BrMADS107*	Bra020242	A02	*AGL62*	248	28.09	1	Mα
108	*BrMADS108*	Bra002480	A10	*AGL62*	279	32.06	1	Mα
109	*BrMADS109*	Bra035685	A04	*AGL40*	293	32.84	3	Mα
110	*BrMADS110*	Bra011938	A07	*AGL23*	238	27.09	1	Mα
111	*BrMADS111*	Bra032057	A04	*AGL61*	180	20.50	0	Mα
112	*BrMADS112*	Bra007829	A09	*AGL61*	207	23.13	0	Mα
113	*BrMADS113*	Bra026764	A09	*AGL62*	168	19.24	0	Mα
114	*BrMADS114*	Bra001209	A03	*AGL91*	179	20.33	0	Mα
115	*BrMADS115*	Bra021910	A04	*AGL29*	182	20.76	0	Mα
116	*BrMADS116*	Bra003884	A07	*AGL60*	212	24.16	0	Mα
117	*BrMADS117*	Bra026674	A09	*AGL100*	206	23.59	0	Mα
118	*BrMADS118*	Bra033492	A01	*AGL84*	293	32.77	0	Mα
119	*BrMADS119*	Bra032767	A04	*AGL84*	309	34.32	2	Mα
120	*BrMADS120*	Bra010027	A06	*AGL73*	345	38.29	0	Mα
121	*BrMADS121*	Bra037434	A06	*AGL73*	261	29.19	0	Mα
122	*BrMADS122*	Bra018727	A06	*AGL74*	245	27.51	0	Mα
123	*BrMADS123*	Bra014217	A08	*AGL84*	277	30.41	0	Mα
124	*BrMADS124*	Bra027116	A09	*AGL55*	243	27.09	0	Mα
125	*BrMADS125*	Bra040965	Scaffold000343	*AGL55*	198	21.96	--	Mα
126	*BrMADS126*	Bra009436	A10	*AGL97*	306	33.85	0	Mα
127	*BrMADS127*	Bra038728	A01	*AGL74*	173	19.84	0	Mα
128	*BrMADS128*	Bra020600	A02	*AGL39*	263	24.32	0	Mα
129	*BrMADS129*	Bra020247	A02	*AGL23*	269	30.64	1	Mα
130	*BrMADS130*	Bra028965	A03	*AGL47*	274	31.46	0	Mβ
131	*BrMADS131*	Bra002611	A10	*AGL82*	297	34.61	0	Mβ
132	*BrMADS132*	Bra037571	A01	*AGL103*	342	39.17	0	Mβ
133	*BrMADS133*	Bra022341	A05	*AGL103*	368	42.12	0	Mβ
134	*BrMADS134*	Bra031864	A02	*AGL52*	331	37.76	0	Mβ
135	*BrMADS135*	Bra025619	A04	*AGL76*	367	42.32	0	Mβ
136	*BrMADS136*	Bra025607	A04	*AGL76*	349	40.12	0	Mβ
137	*BrMADS137*	Bra025609	A04	*AGL76*	336	38.32	0	Mβ
138	*BrMADS138*	Bra018767	A06	*AGL93*	306	34.70	0	Mβ
139	*BrMADS139*	Bra015129	A07	*AGL93*	319	35.90	0	Mβ
140	*BrMADS140*	Bra020923	A08	*AGL89*	209	24.26	0	Mβ
141	*BrMADS141*	Bra018741	A06	*AGL89*	264	30.10	0	Mβ
142	*BrMADS142*	Bra028020	A09	*AGL89*	263	29.76	1	Mβ
143	*BrMADS143*	Bra007138	A09	*AGL89*	281	32.09	0	Mβ
144	*BrMADS144*	Bra028019	A09	*AGL89*	285	32.59	0	Mβ
145	*BrMADS145*	Bra004071	A07	*AGL101*	284	32.33	0	Mβ
146	*BrMADS146*	Bra040248	A01	*AGL46*	413	46.76	1	Mγ
147	*BrMADS147*	Bra005166	A05	*AGL46*	125	14.56	0	Mγ
148	*BrMADS148*	Bra035448	A01	*AGL46*	264	30.77	1	Mγ
149	*BrMADS149*	Bra035449	A01	*AGL46*	264	30.80	1	Mγ
150	*BrMADS150*	Bra039404	A05	*AGL45*	302	34.96	0	Mγ
151	*BrMADS151*	Bra020555	A02	*AGL35*	216	24.32	0	Mγ
152	*BrMADS152*	Bra009913	A06	*AGL35*	203	22.78	0	Mγ
153	*BrMADS153*	Bra018490	A05	*AGL80*	290	33.43	0	Mγ
154	*BrMADS154*	Bra029469	A09	*AGL80*	304	34.48	0	Mγ
155	*BrMADS155*	Bra041022	Scaffold000385	*AGL80*	334	36.92	--	Mγ
156	*BrMADS156*	Bra020552	A02	*AGL37*	341	38.45	0	Mγ
157	*BrMADS157*	Bra020550	A02	*AGL36*	380	42.85	0	Mγ
158	*BrMADS158*	Bra020525	A02	*AGL92*	395	44.76	0	Mγ
159	*BrMADS159*	Bra020524	A02	*AGL92*	360	40.86	0	Mγ
160	*BrMADS160*	Bra009911	A06	*AGL92*	364	41.44	0	Mγ
161	*BrMADS161*	Bra012335	A07	*AGL87*	162	18.99	0	Mγ
162	*BrMADS162*	Bra024521	A09	*AGL87*	162	18.87	0	Mγ
163	*BrMADS163*	Bra028730	A02	*AGL96*	252	28.88	0	Mγ
164	*BrMADS164*	Bra009199	A10	*AGL96*	202	23.13	0	Mγ
165	*BrMADS165*	Bra009176	A10	*AGL96*	192	21.99	0	Mγ
166	*BrMADS166*	Bra009174	A10	*AGL96*	191	22.01	0	Mγ
167	*BrMADS167*	Bra034809	A05	*AGL95*	353	40.27	0	Mγ

### Phylogenetic analysis of MADS-box genes in *B. rapa*

**Figure 1:**
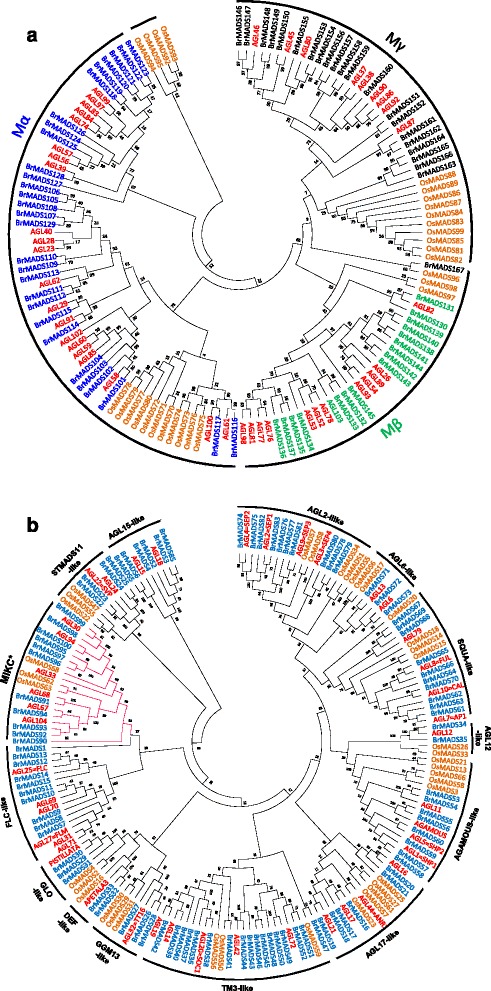
**Phylogenetic tree constructed by the neighbor-joining method using MADS-box genes from** ***B. rapa*** **, Arabidopsis and Rice. (a)** Phylogenetic analysis of 138 type I MADS-box proteins from *B. rapa* (67), Arabidopsis (43) and Rice (28). **(b)** Phylogenetic analysis of type II *B. rapa, R*ice and Arabidopsis MADS-box proteins. 181 type II MADS-box proteins from *B. rapa* (100), Arabidopsis (43) and rice (38) showing 13 MIKC^c^ clades and MIKC* group as marked in the figure.

Independent phylogenetic trees for M-type and MIKC-type MADS-box TFs were constructed using the *B. rapa* MADS-box proteins along with those from Arabidopsis and rice. There were 67 M-type members (i.e., Mα, Mβ and Mγ) from *B. rapa*, with the other 100 proteins belonging to MIKC-type (MIKC^c^ and MIKC^*^; Figure 1). Notably, the MIKC^c^ family included 89 members of this latter group, more than in Arabidopsis, rice, and soybean (Additional file 1: Table S1). Among the 89 MIKC^c^ genes, *BrMADS84, 86, 87, 88* and *89* could not be assigned in the tree using the bootstrap method with 1000 replicates, possibly due to high sequence divergence in the conserved regions and sequence length. To test their relationships and relevance with other MADS-box genes, we generated an alternative phylogenetic tree without using bootstrap replications and found these five genes in the different clades of MIKC^c^ (Additional file 2: Figure S1b).

In accordance with the known classes of Arabidopsis MADS-box genes, we found 13 MIKC^c^ clades in *B. rapa.* Although most of the *B. rapa* MADS-box genes were consistent with Arabidopsis in terms of sequence similarity and grouping, we found some genes *viz. BrMADS41, 47, 167*, that were placed as close sisters of rice MADS-box genes in the tree. Interestingly, *OsMADS59*, instead of being included in the AGL15-like clade, paired with *BrMADS47* in the TM3 clade. There was some disparity in the distribution of rice Mβ genes between the two phylogenetic trees prepared with the different methods (Figure 1a and Additional file 2: Figure S1a). Among the 13 MIKC^c^ clades, the TM3 clade contained the most *B. rapa* sequences (18). The FLC clade included three previously identified *FLC* genes of *B. rapa viz. BrFLC1, BrFLC2, BrFLC3* [40] which showed 99.51, 100 and 100% similarity to *BrMADS13, 12* and *14* respectively at the amino acid level. MIKC*/Mδ included 11 members, which is almost double that in Arabidopsis (6), rice (5) and soybean (5).

In case of type I MADS-box proteins, the Mα and Mγ groups had more members in *B. rapa* (29 and 22 respectively), than in Arabidopsis, rice and soybean. By contrast, the 16 Mβ genes found in *B. rapa* was less than that in Arabidopsis, but more than in rice and soybean (Additional file 1: Table S1) [4,35,38].

### Analysis of conserved motifs in MADS-box proteins of *B. rapa*

**Figure 2:**
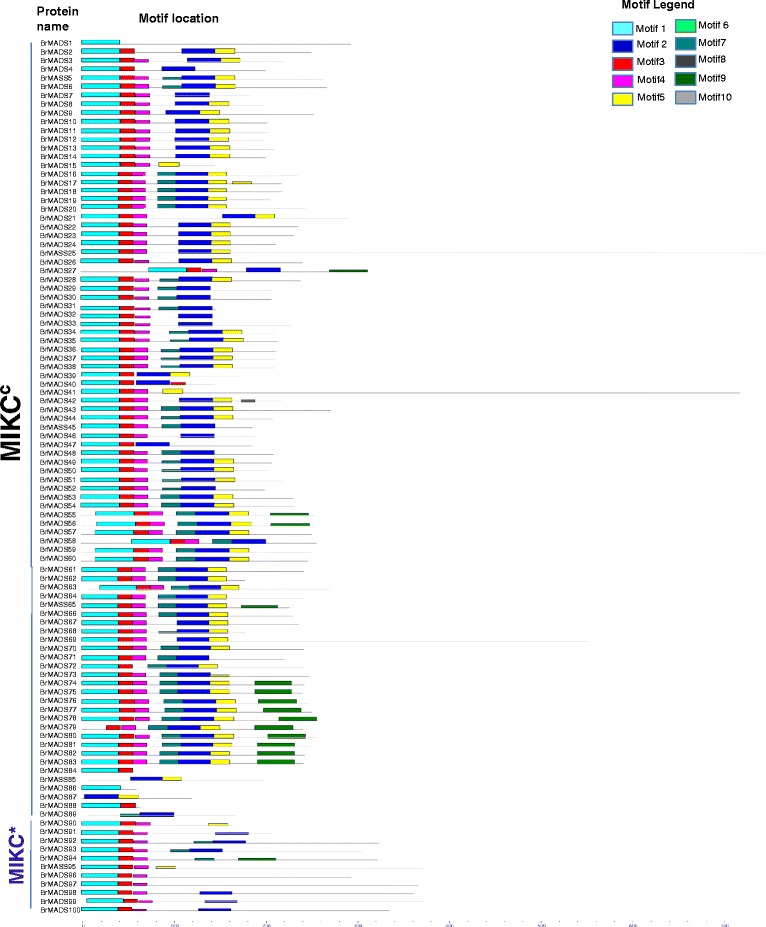
**Schematic representation of motifs identified in** ***B. rapa*** **MADS-box type II proteins using MEME motif search tool for each group (MIKC** ^**c**^ and MIKC*) given separately. Different motifs are indicated by different colors, and the names of all members are shown on the left side of the figure. The order of the motifs corresponds to the position of the motifs in individual protein sequences.

Ten conserved motifs among related proteins were identified from the 167 candidate MADS-box genes of *B. rapa* using the MEME (Multiple Em for Motif Elicitation) motif search tool (Figure 2 and Additional file 2: Figure S3). Motifs 1 and 6 specifying the MADS domain were found in 153 members of the MADS-box family whereas BrMADS79, 85, 87, 89, 105, 109, 113, 118,119, 127, 129, 159, 165 and 167 did not show either motif 1 or 6 characteristic of the MADS domain. These proteins did contain other representative motifs of MADS-box family such as motifs 3, 4, 5, 7, 8, 9 and 10. The MIKC MADS-box proteins exhibited only the motif 1 type MADS domain. Among M-type MADS-box proteins (Mα, Mβ and Mγ), most Mα and Mγ proteins had motif 1-type MADS domains, although BrMADS101 and 102 contained motif 6. Conversely, most of the Mβ proteins (14) had the motif 6-type MADS domain.

Conserved motifs 2, 5 and 7 specified the K domain, which is characteristic of MIKC MADS-box proteins, were found in varying combinations in most MIKC^c^ proteins, except BrMADS1, 84, 86 and 88. MIKC^*^ proteins were found to contain the K-domain motifs (2, 5, and 7) less frequently than did MIKC^c^ proteins (Figure 2). Comparatively less conserved motifs 3 and 4 representative of the I domain were found in both M-type and MIKC MADS-box proteins. Mβ and Mγ type proteins contained I domains at lower frequencies as compared to members of the other groups. A considerable number of non-MIKC proteins, especially from the Mα group, showed partial K domain motifs. Finally, motifs 8, 9 and 10 representing the C-terminal domains were also weakly conserved among *B. rapa* MADS-box genes. Motif 9 was restricted to 14 MIKC^c^ and 1 MIKC* proteins. All Mγ proteins except BrMADS161 and 162 consistently showed both the C-terminal-representing motifs 8 and 10. Motif 8 and 10 were limited to only M-type MADS-box proteins. The Mα group showed motif 8, but motif 10 was exclusively present in the Mγ proteins. The Mβ group showed an interesting pattern, wherein 7 genes contained only a single motif, specifically one representative of the ‘MADS’ domain. Only 4 Mβ genes out of 16 had more than two full or partial motifs (Additional file 2: Figure S3).

### Syntenic relationships between MADS-box genes of *B. rapa* and Arabidopsis

Polyploidy [arising from whole-genome duplication (WGD)] has played a vital role in the evolution and genetic diversity of angiosperm genomes [41]. WGD events are generally followed by changes in gene expression and widespread gene loss [42]. The *Brassica* genus is closely related to the model species *A. thaliana* and both are members of the *Brassicaceae* family. Comparative genetic and physical mapping as well as genome sequencing studies have authenticated the syntenic relationships between the Arabidopsis genome and the triplicate genome of *B. rapa*, with subgenomes having evolved by genome fractionation [43,44]. Comparative analysis was conducted to identify homologous MADS-box transcription factors between *B. rapa* and Arabidopsis. Based on our phylogenetic results and BLASTX reconfirmation, we determined which Arabidopsis MADS-box genes were orthologous to the 167 MADS-box *B. rapa* homologs. Among the homologous gene sets, we found that most Arabidopsis MADS-box genes were represented by one to three copies of *B. rapa* MADS-box genes (Additional file 1: Table S3).

### Chromosomal location of MADS-box genes and their genomic duplication in *B. rapa*

**Figure 3:**
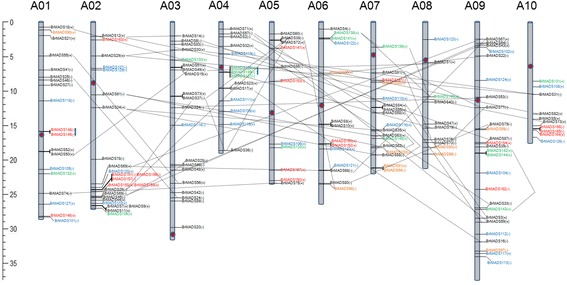
**Chromosomal location of** ***B. rapa*** **MADS-box genes along ten (10) chromosomes.** Respective chromosome numbers are written as A01 to A10 on the top of each chromosome. Different colors of gene name represent different groups (black: MIKC^c^, orange: MIKC*, blue: Mα, green: Mβ and red: Mγ). The positive (+) and negative (−) signs following each gene represent forward and reverse orientation of the respective gene. Genes lying on duplicated segments of genome are joined by black dotted lines. Tandemly duplicated genes are shown by blue vertical blue lines. Gene position and each chromosome size can be estimated using the scale (in Megabase; Mb) on the left of the figure.

We mapped the physical locations of the MADS-box genes on the 10 chromosomes of *B. rapa* (except two genes mapped to scaffolds *Scaffold000343* and *Scaffold000385*; Figure 3). The highest numbers of MADS-box genes were found on chromosomes 9 (26 genes; 15.8%) and 2 (24 genes; 14.5%), while chromosomes 8 and 10 contained the fewest (10 each). Among the five types of MADS-box genes, MIKC* and Mγ genes were clustered along chromosomes 1, 6, 7, 8, 9 and chromosomes 1, 2, 5, 6, 7, 9, 10, respectively. A high of 18 MIKC^c^ genes was found on chromosome 3, but other than that there was no bias was observed in the distribution of MIKC^c^, Mα or Mβ genes (Figure 3). Duplication analysis revealed that 67 out of 167 MADS-box genes (40.12%) were present in two or more copies. This gene duplication occurred as a result of tandem and segment duplications. A total of 63 MADS-box genes were found to have counterparts on duplicated segments. We observed, higher frequencies of segmental duplications generated many homologs of MADS-box genes along all chromosomes of *B. rapa* (black dotted lines in Figure 3). Conversely, lower frequencies of tandem duplications were evident among M-type *B. rapa* MADS-box genes. Only 4 tandemly duplicated genes (from Mβ and Mγ) were found on chromosomes 1 and 4. Evolutionary analysis of *B. rapa* also validated our findings, wherein only 14% of the *B. rapa* genes were tandem duplicates, compared with 27% of Arabidopsis genes in a 100-kbp window interval [45]. No large gene clusters or hot spots for *B. rapa* MADS-box genes were identified, possibly due to the very few tandem duplications.

### Transcript analysis of *B. rapa* MADS-box genes during organ development

**Figure 4:**
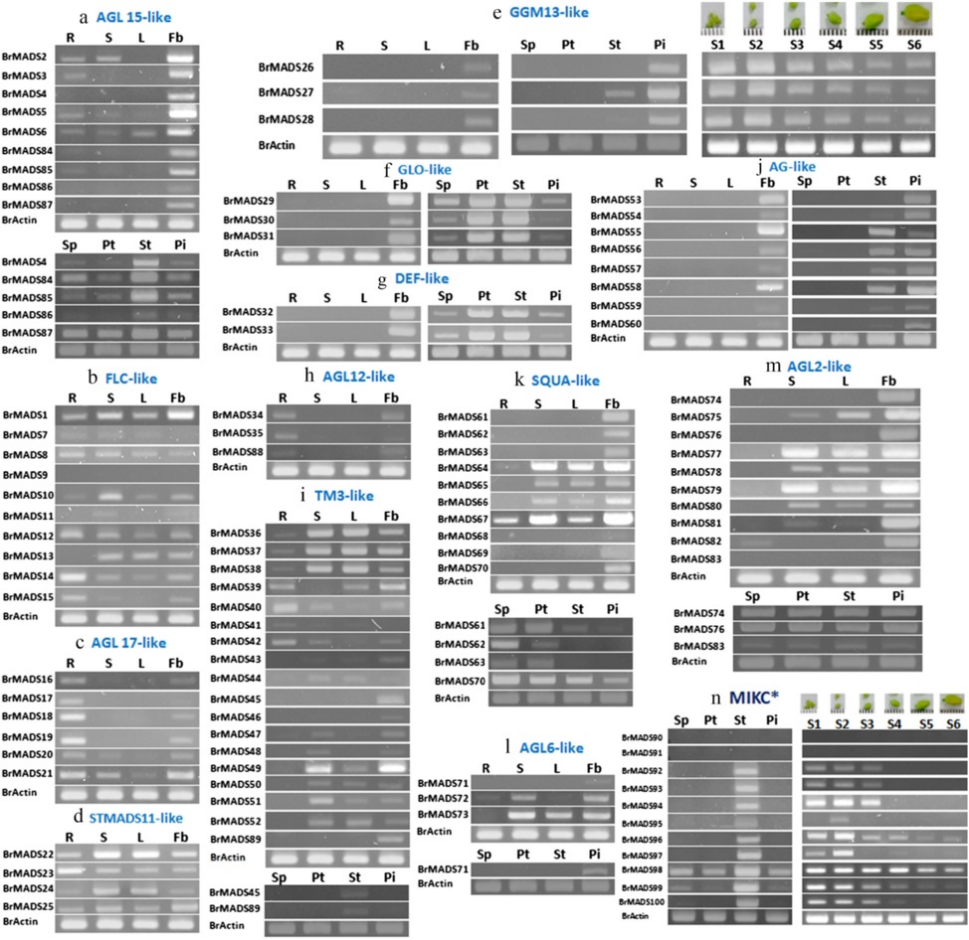
**Organ specific expression analysis of 100** ***MIKC BrMADS*** **according to phylogenetic grouping (a-n) are showing in the root (R), stem (S), leaf (L), flower bud (Fb), sepal (sp), petal (pt), stamen (st), pistil (pi) and six flower growth stages of** ***B. rapa*** **(young to mature buds are marked as S1 to S6 on the top of the figure).**

MADS-box genes have been found to be involved primarily in floral organ specification; although some recent studies revealed their involvement in other processes as well. Specifically, MIKC^c^ proteins among all the MADS-box groups have been found to have diverse functions related to plant growth and development [1,25,35,46]. We therefore examined the expression of all 89 *B. rapa* MIKC^c^ genes in root, stem, leaf and flower buds. We also investigated these genes in the sepal, petal, stamen and pistil of *B. rapa* flower which had expressions only in the flower buds. And, we discussed the expression of all MIKC^c^ genes here in accord with thirteen clades identified in our study. Additionally, we included all MIKC* genes in the four floral tissue expression study as they have been reported to be involved in the development of reproductive organs [47]. Finally, we conducted an expression study in six flower bud developmental stages (young to mature bud stage) for selected MIKC^c^ genes (those expressed only in flower buds) and all MIKC* genes to justify their roles during the flower bud development (Figure 4).

#### AGL15-like genes

It has been reported that *AGL15* in Arabidopsis strongly delays abscission and senescence in reproductive tissues [9]. The *B. rapa* genome has nine AGL15-like genes (*BrMADS2, 3, 4, 5, 6, 84, 85, 86, 87*) and their expression in different tissues was consistent with that of their closest Arabidopsis homologs. All of the genes had predominant expression in flower buds while a few of them were expressed at low levels in different vegetative tissues (Figure 4a).

#### FLC-like genes

*FLC* acts as an inhibitor of flowering and is a convergence point for environmental and endogenous pathways that regulate flowering time in Arabidopsis [9]. We found ten *FLC* homologs [*BRMADS1, 7, 8, 9, 10, 11,* and *15* in addition to the previously identified *BrFLC1* (*BrMADS12*), *BrFLC2* (*BrMADS13*), and *BrFLC3* (*BrMADS14*)] in *B. rapa* with very similar expression patterns in most organs. *BrMADS1* is a distant member of this subfamily and showed strong expression in the four tissues tested. Our root expression results for *BrFLC1* and *BrFLC2* contrast with those previously reported [40]. This might be due to varietal differences of *B. rapa* between the two studies. *BrMADS9* is the only member of this subfamily that was not expressed in any of the organ tissues (Figure 4b).

#### AGL17-like genes

The AGL17-like genes show unusually diverse expression patterns, with members being expressed in roots (majority of genes), in pollen (*DEFH125* in *Antirrhinum*), in both (*ZmMADS2* in maize), or in leaf guard cells and trichomes (*AGL16*) [9]. We identified six AGL17-like genes (*BrMADS16, 17, 18, 19, 20*, *21*) and found expression primarily in roots of *B. rapa* like their Arabidopsis counterparts*.* Additionally, they were expressed in flower buds like in other eudicots [9]. We also observed low expression in stem and leaf tissues (Figure 4c).

#### STMADS11-like genes

Genes of this clade perform contrasting roles in flower development. *SVP* (*SHORT VEGETATIVE PHASE*) functions as a floral repressor, whereas *AGL24* belongs to the same subfamily but promotes flowering in Arabidopsis [48,49]. We identified four genes (*BrMADS22, 23, 24, 25*) in this subfamily and detected their widespread expression in the four organs of *B. rapa* (Figure 4d). This is in contrast to the expression of *SVP* in Arabidopsis, which is restricted to leaves and shoots [9].

#### GGM13-like genes

The GGM13-like genes are expected to represent a sister group of the B genes and hence are termed B_sister_ (B_s_) genes [9]. *ABS/TT16* is the only Arabidopsis GGM13-like gene and has been shown to function in the specification of endothelial cells as well as in the control of flavonoid biosynthesis in the seed coat [23]. We identified three GGM13-like genes (*BrMADS26, 27*, *28*), with expression exclusively in the flower buds like their Arabidopsis counterparts. All three were expressed in the female reproductive organ of *B. rapa* flowers, whereas *BrMADS27* was also expressed in the male reproductive organ. Interestingly, transcript accumulation of all GGM13-like genes gradually decreased from early to mature bud stage of flower development (Figure 4e).

#### *GLO* and *DEF-like genes*

These genes are B class floral homeotic genes in eudicots and are involved in specifying petals and stamens during flower development [50]. We found three GLO-like genes (*BrMADS29, 30, 31*) and two DEF-like genes (*BrMADS32, 33*) that were expressed exclusively in the flower buds. Transcripts for these genes were abundant in the petals and stamens of *B. rapa* flowers. We also found low expression in sepals and pistils (Figure 4f & 4g).

#### AGL12-like genes

Three AGL12-like genes (*BrMADS34, 35*, *88*) with preferential expression in roots were detected in *B. rapa. BrMADS34* and *88* were also expressed in the flower buds, similar to their Arabidopsis counterpart *AGL12* with the exception that *AGL12* has also been detected in shoots (Figure 4h)*.*

#### TM3-like genes

These genes are expressed preferentially in vegetative parts of other plant species [51,52]. *SOC1* is an important member of this family expressed abundantly in the apical meristem and acting as a flowering time regulator [53]. We identified eighteen TM3-like genes (*BrMADS36, 37, 38, 39, 40, 41, 42, 43, 44, 45, 46, 47, 48, 49, 50, 51, 52* and *89*) with variable expression patterns in vegetative and reproductive parts of *B. rapa. BrMADS36, 37* and *38* are close homologs of *SOC1* and were primarily expressed in stem, leaf and flower buds. Moreover, we found *BrMADS39, 40* and *42* to be expressed primarily in roots, but unlike their Arabidopsis counterparts (*AGL14* and *AGL19*), we detected their expression in other parts of the plant as well (Figure 4i).

#### AG-like genes

Genes of this clade are mainly involved in specifying stamen and carpel identity, and in providing floral determinacy [9]. We identified eight AGAMOUS-like (AG) genes (*BrMADS53, 54, 55, 56, 57, 58, 59, 60*) that were expressed exclusively in flower buds of *B. rapa*. Our results are consistent with those for the Arabidopsis *AG* subfamily, members of which specify stamen and carpel identity [54]. Some of these *B. rapa* genes were pistil specific (*BrMADS53* and *54*) and some were expressed in both male and female reproductive organs (*BrMADS55, 56, 57, 58, 59* and *60*) (Figure 4j).

#### SQUA-like genes

SQUA-like genes are typically expressed in inflorescence or floral meristems, and most of them function as meristem identity genes [9]. In addition, they are involved in specifying sepals and petals and thus are class ‘A’ floral organ identity genes [55]. We identified ten SQUA-like genes (*BrMADS61, 62, 63, 64, 65, 66, 67, 68, 69, 70*) that had variable transcript patterns, but were expressed mainly in flower buds like their Arabidopsis counterparts. Some *BrMADS* SQUA-like genes showed strong expression in the stem and leaf as well. Our results in this case are also consistent with the Gu *et al*. findings, where they detected the SQUA-like gene ‘*FRUITFULL’* in stems and leaves of Arabidopsis [21]. *BrMADS67* was the only member of this subfamily expressed in all tested organ tissues of *B. rapa* (Figure 4k).

#### AGL6-like genes

The functions of AGL6-like genes are not clear. We isolated three AGL6-like genes (*BrMADS71, 72, 73*) from *B. rapa* with expression in the flower buds, like their Arabidopsis counterparts *AGL6* and *AGL13. BrMADS72* and *73*, unlike their close homolog *AGL6*, also showed expression in vegetative tissues (Figure 4l).

#### AGL2-like genes

These genes play a central role in the floral meristem and floral organ development [56]. They constitute an additional class of floral homeotic genes, termed as class E genes [9]. Ten AGL2-like (*BrMADS74, 75, 76, 77, 78, 79, 80, 81, 82, 83*) genes from *B. rapa* showed expression primarily in reproductive tissues. *BrMADS75, 77, 78, 79, 80* and *81* were also expressed in the stem and leaf, and *BrMADS82* alone had additional very low expression in roots (Figure 4m).

#### BrMIKC* genes

There were eleven MIKC* genes (*BrMADS90, 91, 92, 93, 94, 95, 96, 97, 98, 99, 100*) that were placed apart from the other MIKC genes in the phylogeny. Most of these genes were found to be expressed exclusively in the stamens, except in the case of *BrMADS98* and *99*, that were detected in the four floral organ tissues. Moreover, these genes showed differential expression in six flower bud developmental stages (young to mature bud stage). *BrMADS96, 98, 99* and *100* were preferentially expressed in the young bud stage while their expression gradually decreased until to the mature bud stage. The rest of the genes exhibited widespread expression mainly in the early stages of bud development. However, two MIKC* genes (*BrMADS90* and *91*) appeared to be nonfunctional, as they were not expressed in any stage of bud development or in any floral organ tissues (Figure 4n).

### Microarray expression against cold and freezing stress

**Figure 5:**
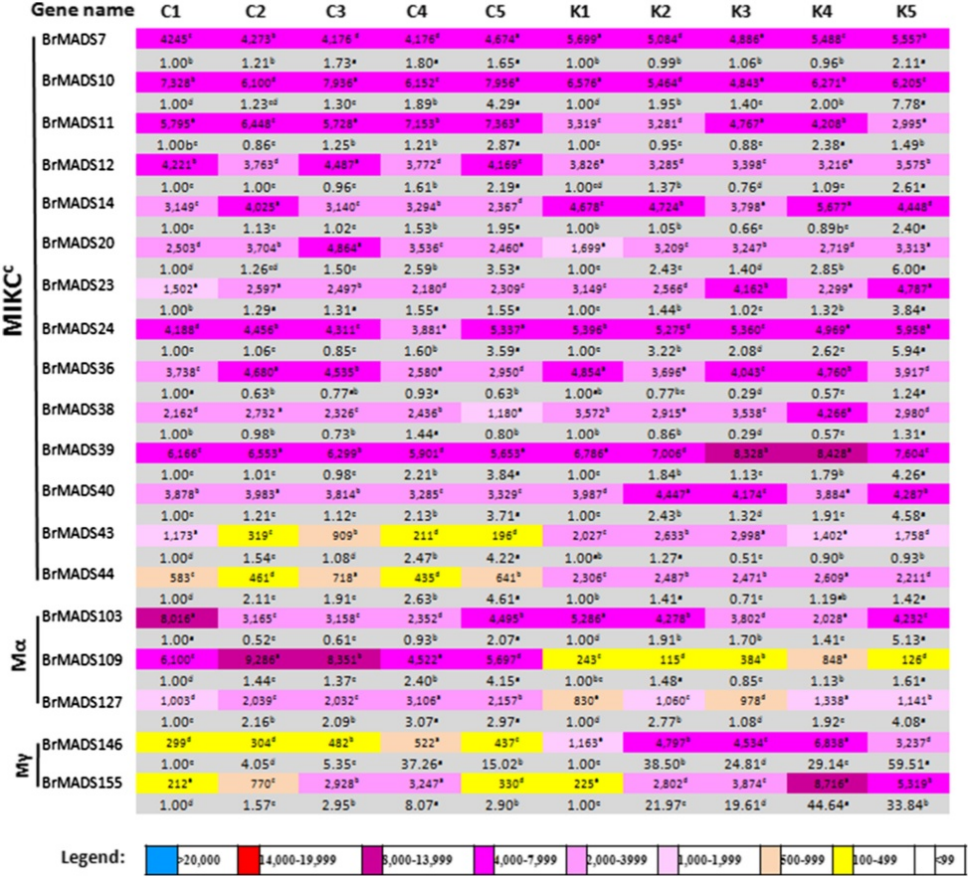
**Microarray (upper colored rows) and qPCR expression (lower grey colored rows) against each 19 MADS-box genes in** ***B. rapa*** **under control (C1&K1), 4°c (C2&K2), 0°c (C3&K3), −2°c (C4&K4), and −4°c (C5&K5) temperature treatments.** Here C and K stand for ‘chiifu’ and ‘kenshin’ two inbred lines of *B. rapa* respectively. Responsive genes in different temperature from different MADS-box groups have been shown on the left side. Color bar at the base representing differential expression in microarray. Values denoted by the same letter did not differ significantly at P < 0.05 according to Duncan’s multiple range tests.

Four weeks old seedlings of two inbred lines of *B. rapa,* Chiifu and Kenshin, were treated with cold and freezing stresses (4°C, 0°C, −2°C and −4°C) during 2 hours and the expression of the 167 MADS-box genes were subsequently analyzed using microarrays*.* Chiifu originated in temperate regions, whereas Kenshin originated in subtropical and tropical regions and therefore, these two lines are expected to respond differently against cold and freezing stresses. Only 19 MADS-box genes from different groups showed differential cold- or freezing-responsive expression between the two lines (Figure 5), while the remaining 148 genes showed very low or no expression (Additional file 2: Figure S4). Among the 19 differentially expressed genes, 14 MIKC^c^ genes showed varying levels of expression, with *BrMADS7, 10, 24* and *39* displaying similar expression patterns in response to cold and freezing. *BrMADS11, 12, 14, 20, 23, 36, 38* and *40* were expressed at different levels than the aforementioned four MIKC^c^ genes in both lines of *B. rapa. BrMADS43* and *44,* two MIKC^c^ genes, were expressed at low levels in Chiifu throughout the stress period, while in Kenshin they showed constitutive expression. By contrast, three genes from the Mα group (*BrMADS103, 109* and *127*) showed differential expression within and between the two lines, with Chiifu exhibiting higher expression than Kenshin. Notably, two Mγ genes (*BrMADS146* and *BrMADS155*) showed higher responsiveness in Kenshin than in Chiifu upon exposure to cold and freezing temperatures (Figure 5).

### qPCR expression of MADS-box genes against abiotic stress

**Figure 6:**
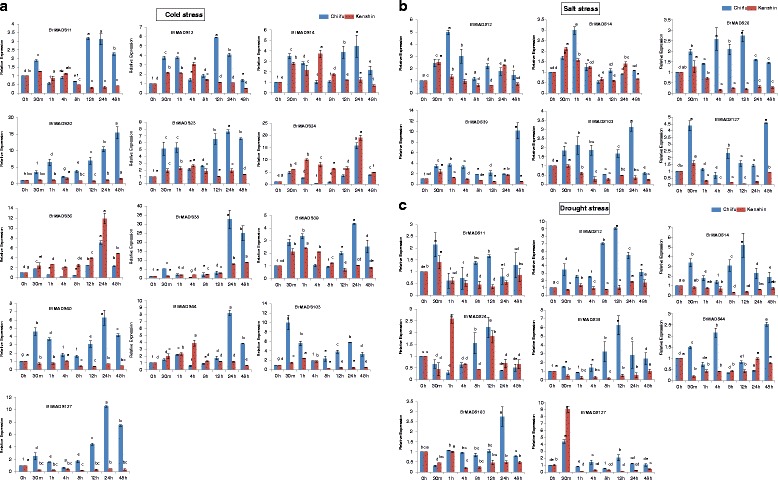
**Real-time PCR expression analysis of MADS-box genes after cold, salt and drought stress treatment (0-48 h) in** ***B. rapa*** **(a-c)** ***.*** The error bars represent the standard error of the means of three independent replicates. Values denoted by the same letter did not differ significantly at P < 0.05 according to Duncan’s multiple range tests.

One of our main objectives was to identify MADS-box genes that might show stress responsiveness in addition to having different growth functions. At first, a qPCR experiment was conducted to validate the cold and freezing responsiveness of the 19 *BrMADS* genes which were selected from the microarray analysis. We observed their expression patterns and found them consistent with the microarray results in most of the cases. Only two genes (*BrMADS43* and *44*) were found to show their expressions differently from those in the microarray experiment (Figure 5). However, for a better understanding of gene expression in response to three abiotic stresses (cold, salt and drought) in a time course basis (0 h, 30 min, 1 h, 4 h, 8 h, 12 h, 24 h and 48 h) we again selected two inbred lines of *B. rapa*, Chiifu and Kenshin. Leaf and root tissues of stress treated *B. rapa* were examined for qPCR expression analysis. Besides cold stress, we also examined the salt and drought responsiveness of the same MADS-box genes. Arora *et al.* found MADS-box genes involved in responses to multiple stresses [35]. The 19 differentially expressed MADS-box genes from the whole-genome low temperature-treated data set were selected for qPCR experiments (Additional file 2: Figure S4 and Figure 5). In Chiifu, *BrMADS11, 12, 14, 20, 23, 24, 36, 38, 39 40, 44, 103* and *127* showed differential expression in response to cold stress, wherein they were up-regulated from 0 h to 1 h and down-regulated at 4 h-8 h. Subsequently, all genes were up-regulated from 8 h to 24 h and exhibited their highest expression at 24 h (except *BrMADS20*, which showed the highest expression at 48 h), followed by a down-regulation at 48 h. Apart from these, *BrMADS103* showed the highest expression at 30 m, after which it followed the same expression patterns as the others. Conversely, in Kenshin, *BrMADS11, 12, 14, 23, 39*, *44* and *103* were up- regulated at early hours of stress after which they showed down-regulation and eventually became inactive at later stages of cold stress. *BrMADS24,* and *36* in Kenshin exhibited 19- and 12-fold higher expression respectively than the control throughout the stress period and, more interestingly, expression of these two genes in Chiifu was far below that in Kenshin. Notably, from the thirteen cold responsive *BrMADS* genes eleven were form MIKC^c^ group. More specifically, among these genes, three (*BrMADS11, 12* and *14*) were from FLC-like clade, one (*BrMADS20*) from AGL17-like clade, two (*BrMADS23* and *24*) from STMADS-like clade and five (*BrMADS36, 38, 39, 40* and *44*) from TM3-like clade (Figure 6a).

During salt stress, *BrMADS12, 14, 39*, *103* and *127* in Chiifu were up-regulated up to 1 h, showed down-regulation in the mid-stage of stress and were up-regulated again at later stages. *BrMADS20* was alternatively up and down-regulated up to 12 h and afterwards it showed down-regulation from 24 h - 48 h. In Kenshin, these same six MADS-box genes were induced early in salt treatment (up to a maximum of 2-fold in *BrMADS12* and *39*) and down-regulated for the rest of the period (Figure 6b).

In the case of drought stress, *BrMADS11, 12, 14, 24, 38, 44, 103* and *127* were expressed differentially in both Chiifu and Kenshin. Six genes (*BrMADS11, 12, 14, 38, 44* and *127*) in Chiifu were up-regulated at 30 m after administering drought stress, while *BrMADS11, 12, 14* and *38* were down-regulated from 4 h - 8 h. *BrMADS24* and *103* were down-regulated at early stage, after which *BrMADS24* was up-regulated from 4 h - 12 h and down-regulated again from 24 h - 48 h. After 30 m, *BrMADS103* remained static except at 24 h when it was induced more than 2 fold. By contrast, these six MADS-box genes in Kenshin were down-regulated soon after drought treatment and remained that way throughout the stress period. Though *BrMADS11, 24* and *127* showed up-regulation at an early stage, they eventually became inactive for the rest of the period (Figure 6c).

## Discussion

### Duplication among MIKC genes seems to have played major role in the expansion of MADS-box genes in *B. rapa*

In this study, we have reported 167 MADS-box genes of *B. rapa*, which is higher in number than the MADS-box genes in Arabidopsis (107) [4]. The whole genome of *B. rapa* underwent triplication events since its divergence from Arabidopsis [32]. Thus, evolutionary relationship between *B. rapa* and Arabidopsis is also supportive to our findings. On the other hand, we observed the expansion of MIKC and M-type genes in these two linages. We found some disparity on the duplication events between the MIKC and M-type genes of *B. rapa* and Arabidopsis. For example, duplication events took place with higher frequency among MIKC-type *B. rapa* MADS-box genes compared to M-type genes. And, in case of Arabidopsis this scenario was reverse, where more number of M-type genes than MIKC genes was found in the duplicated segments. More specifically, 57 MIKC genes were found in duplicated segments of *B. rapa* (black dotted lines in Figure 3). This might be related to the fact that there are more pseudogenes of M-type than of MIKC-type MADS-box genes in the Arabidopsis genome and they experienced faster birth and death rates than MIKC type [57]. Although the *B. rapa* genome is triplicated relative to that of Arabidopsis, the number of M-type genes in *B. rapa* is almost the same as in Arabidopsis (Additional file 1: Table S1). We speculate this might be due to the presence of many non-functional M-type genes (i.e., psuedogenes) that remained inactive and were not duplicated or were deleted from the *B. rapa* genome. MIKC-type genes have functioned in growth and development of plants since their evolution and after multiple duplication events in *B. rapa*, MIKC-type genes appear to have functionally differentiated in a relatively short time and been maintained as functional genes in the genome to perform more complex functions flower and organ development.

### Involvement of MADS-box genes in organ development of *B. rapa*

#### Role in reproductive organ development

Investigations regarding the genetic and molecular basis of floral development in the model eudicots Arabidopsis and *Antirrhinum* have revealed the involvement of a number of MADS-box genes in specifying floral organ identity [58]. The high degree of sequence identity and remarkably conserved genome structure between Arabidopsis and *Brassica* genomes enables comparison of crop genomics among the *Brassica* complex [45]. In this study, we investigated the Arabidopsis MADS-box homologs in *B. rapa* that play specific roles in flower development. Consideration of the ABCDE model of flower development in *B. rapa* revealed extensive similarities with that of Arabidopsis and other higher plants.

All SQUA-like genes in *B. rapa* were typically expressed in the flower buds like their Arabidopsis counterparts. *AP1* is involved in specifying sepals and petals as class A floral organ identity gene [53]. Our results also suggest that *BrMADS61, 62,* and *63* as putative orthologs of *AP1* might play similar role, and they have sepal- and petal-specific expression in *B. rapa* flowers (Figure 4k).

Regarding the B class genes in *B. rapa*, we found five close homologs of Arabidopsis *PISTILLATA* (*PI*) and *APETALA3* (*AP3*) that showed distinct expression in male reproductive organs but not female reproductive organs. Besides being involved in the male and female reproductive parts, these genes were also recruited for petal identity in Arabidopsis [59]. We also found petal expression for them in *B. rapa* flowers.

Genes involved in C and D functions are from the monophyletic AG subfamily. All *AG* family genes in *B. rapa* had higher expression in female organs than in male. C and D class genes like *STK/AGL11, SHATTERPROOF1* (*SHP1*)*,* and *SHP2*, are together required for ovule identity [52]. Close homologs of *SEP* (*SEPALLATA*) genes from the AGL2-like subfamily in *B. rapa* showed widespread expression mainly in the aboveground parts; this is suggestive of their involvement in organ development. Pelaz *et al.* studied triple mutants of Arabidopsis *SEP* family genes (*SEP1, SEP2* and *SEP3*) and found that their redundant functions are required for petal, stamen and carpel development and to prevent indeterminate growth of the flower meristem [20]. Genes of this family have been identified in fruits during the ripening stage of grapevine [13]. Similarly, two tomato *SEP* genes, *TM29* and *LeMADSRIN*, appear to play roles in tomato fruit development [60]. The AGL12 subfamily has three members in *B. rapa*, two in poplar and one each in Arabidopsis and grapevine. Genes from this subfamily have found to play roles in the regulation of cell cycle in root meristems and as promoters of flowering transition through up-regulation of *SOC1, FLOWERING LOCUS T* (*FT*) and *LEAFY* (*LFY*) [27].

We found both reproductive and vegetative expression of AGL15 subfamily genes in *B. rapa*, as in Arabidopsis, whereas they were restricted to the flower buds, flowers and fruits in grapevine [13]. *AGL15* and *AGL18* are proposed to function as repressors of floral transition, acting upstream of *FT* and probably in combination with other floral repressors like *SVP* or *FLC* [61]. Our results regarding AGL17*-*like genes correspond with their expression in Arabidopsis, where they are expressed primarily in roots, which indicate that they might function in *B. rapa* root development. The flower bud expression of the AGL17*-*like genes in *B. rapa* is also consistent with the assumption of a flowering promoter role for *AGL17*, which could participate in the photoperiodic induction of *AP1* and *LFY* independent of *FT* [62].

Predominant expression of *B. rapa* MIKC* genes in the young bud stage demonstrates their importance in male reproductive organ development. Our results contrast with those for AtMIKC*, for which Verelst *el al.* reported predominant expression during late stages (mature pollen grain stage) of pollen development [47].

Predominant expression of three *TT16* homologs (*GGM13-like genes*) in the early stage of female reproductive growth demonstrates their importance in the development of this organ (Figure 4e). These findings are similar to that of a previous investigation in Arabidopsis*,* where GGM13-like gene expression was observed in female reproductive organs, especially in ovules, which is also consistent with the situation in gymnosperms and other angiosperms [63]. Moreover, *TT16* from Arabidopsis is the only *GGM13*-like gene for which a mutant phenotype is known. Analysis of this mutant revealed that *TT16* is involved in the specification of endothelial cells and control of flavonoid biosynthesis in seed coat [23].

#### Role of MADS-box genes in vegetative tissue development

Transcription of a number of MADS-box genes outside flowers and fruits as well as an increasing number of mutant and transgenic flowering plants suggest that members of this gene family play regulatory roles during vegetative development also, such as in embryo, root and leaf development [1,10]. The existence of MADS-box genes in gymnosperms, ferns, and mosses, which do not form flowers or fruits, further demonstrates the role of these genes in plants is not restricted to flower or fruit development [12,64].

All homologs from the AGL17-like clade in the *B. rapa* genome were predominantly expressed in roots and some of them were detected in stem and leaf tissues as well. Reports from different studies indicate that AGL17-like genes show unusually diverse expression patterns in roots, pollen, leaf guard cells and trichomes. It is likely that the ancestral AGL17-like gene had an expression domain restricted to vegetative tissues [1].

In Arabidopsis*, AGL18* and *AGL15* showed high expression in roots, flowers, siliques, and significant expression was also observed in stem and leaves. Moreover, *AGL18* was detected up to the heart stage of embryo development but not in the developing embryos at any stage [1]. Accordingly, we can also predict that *BrMADS2, 3, 4* and *85* in *B. rapa*, as putative orthologs of *AGL18*, might play roles in vegetative tissue development.

TM3-like genes in Arabidopsis (*AGL14* and *AGL19*) have been reported to function in the roots (in the columella, lateral root cap, and epidermal cells of the meristematic region and in the central cylinder of the mature roots) [1,13]. *SOC1*, a floral pathway integrator, expressed most abundantly in aboveground parts, is repressed by another MADS-box gene, the floral transition repressor *FLC,* which is involved in vernalization [65,66].

The ubiquitous expression of some *B. rapa FLC* genes corresponds to that of their Arabidopsis homologs. Kim *et al.* reported that the expression of three *BrFLC* genes (*BrFLC1, BrFLC2, BrFLC3*) was associated with flowering time and concluded that *BrFLC* genes act similarly to *AtFLC* and ultimately help in controlling of flowering time in *B. rapa* and other crops as well to produce higher vegetative yields [40].

The ubiquitous expression of *B. rapa* STMADS11-like genes suggests that these might be good candidates to play regulatory roles. Reports on *STMADS11* genes from different crops demonstrated that they play important roles in developing vegetative tissues. For example, *JOINTLESS*, a tomato (*Solanum lycopersicum*) MADS-box gene is required for the development of a functional abscission zone in tomato flowers [67]. Transcripts of the potato MADS-box genes *STMADS11* and *STMADS16* are present in all vegetative tissues of potato, including roots and new tubers, but are not detected in floral organs [68].

*BrMADS SQUA-like* genes expressed in the vegetative tissues might have some regulatory roles related to vegetative tissue development. *Potato* MADS*-box 1 (POTM1)* a potato *SQUA-like* gene, exhibited widespread expression in actively growing tissues such as meristems, roots, new leaves and new tubers [69].

### Stress responsive MADS-box genes in *B. rapa*

MADS-box genes have already been identified to play roles under low temperature stress in tomato [70], while seven MADS-box genes have been demonstrated to take part in stress (cold, salt and drought) responses in rice [35]. Our qPCR analysis revealed differential expression of thirteen MADS-box genes (*BrMADS11, 12, 14, 20, 23, 24, 36, 38, 39, 40, 44, 103,* and *127*) in response to cold stress (Figure 6a). We observed, expression patterns some of these potential genes (*BrMADS23, 24, 36, 38, 44* and *103*) were not consistent with the microarray results. However, we identified some candidate stress-resistance and stress-susceptibility genes based on up- and down-regulation of the genes between two inbred lines, Chiifu and Kenshin, of *B. rapa*. We found that Chiifu, as a cold-resistant line, showed more up-regulation of MADS-box genes than did Kenshin in response to cold stress via qPCR analysis. The exceptions were *BrMADS24* and *36*, which exhibited much higher up-regulation in Kenshin than in Chiifu and these two genes might be related to cold susceptibility in Kenshin. The highly expressed MADS-box genes in Chiifu might be involved in cold resistance, while their inactivity or very low activity in Kenshin might play a role in the cold susceptibility of that line. We also identified six (*BrMAD12, 14, 20, 39,103,* and *127*) and eight (*BrMADS11, 12, 14, 24, 38, 44, 103,* and *127*) MADS-box genes as differentially expressed in response to salt and drought, respectively (Figure 6b & 6c). Similar phenomena as in cold stress were also observed in case of resistance against salt and drought stresses between the two lines of *B. rapa*. Finally, we found *BrMADS12, 14, 103* and *127* to be co-responsive against all three stresses, suggesting that these genes might have multiple stress resistance related functions in *B. rapa*. Among the stress-induced genes, eleven were from the important MIKC^c^ group, which is well known for regulatory roles in growth and development of different higher plants. *FLC* is repressed by cold and others *FLC-like* genes are also responsive to temperature in different ways [71]. We also identified three cold responsive *B. rapa FLC-like* genes (*BrMADS11, 12* and *14*) from this clade. In rice, all seven stress-responsive genes were also from MIKC^c^ [35]. Likewise, in wheat, a large number of genes involved in flower development are associated with abiotic stress responses [34]. Moreover, we found two Mα genes (*BrMADS103* and *127*) to show stress responsiveness in *B. rapa*, which has not been reported in any plant yet. Our findings here serve as an important resource guiding specific investigations on the stress resistance of *B. rapa* related to MADS-box genes.

## Conclusion

This is a comprehensive and systemic analysis of MADS-box TFs in *B. rapa* wherein we demonstrated their expression patterns in different growth organs and examined their responses to various abiotic stresses as well. Our data set presented here, which includes likely B and C function genes that display male organ-specific expression, should be an important resource for study of male sterility in *B. rapa*. Furthermore, the stress-responsive genes described in this study might be exploited for molecular breeding of *B. rapa.* The results presented here also facilitate selection of appropriate candidate genes for further functional characterization.

## Methods

### Identification of MADS-box genes

A search of SWISSPROT annotations at the *Brassica* database (BRAD) was conducted using keyword ‘MADS-box’ (http://brassicadb.org/brad/) [37]. Protein and CDS of the resulting candidate *B. rapa* MADS-box genes were obtained from the *Brassica* database (http://brassicadb.org/brad/) [37]. To confirm the presence of a MADS-box domain, the web tool from EMBL (http://smart.embl.de/smart/set_mode.cgi?GENOMIC=1) and homology searches using the Basic Local Alignment Search Tool (BLAST; http://www.ncbi.nlm.nih.gov/BLAST/) were performed on the set of candidate MADS-box genes in *B. rapa*. The primary structure of the genes was analyzed using protParam (http://expasy.org/tools/protparam.html). The number of introns and exons was determined by manually aligning the CDS sequences with the genomic sequences using ClustalW [72] and with the ‘Gene Structure Display Server’ (GSDS) web tool [73].

### Phylogenetic analysis of MADS-box proteins

*B. rapa* MADS-box proteins were aligned using ClustalX with those of rice and Arabidopsis. [74]. The phylogenetic trees were generated with MEGA6.06 using the Neighbor –Joining (NJ) algorithm [75]. Bootstrap analysis with 1,000 replicates was used to evaluate the significance of the nodes. Pairwise gap deletion mode was used to ensure that the divergent domains could contribute to the topology of the NJ tree. For generating alternative phylogenetic trees all the protein sequences were aligned in ClustalW using default parameters [72] and the phylogenetic trees were constructed using MEGA6.06 [75].

### Analysis of conserved motifs in MADS-box proteins

The MADS-box protein sequences were analyzed using the MEME software (Multiple Em for Motif Elicitation, V4.9.0) [76]. A MEME search was executed with the following parameters: (1) optimum motif width ≥6 and ≤200; (2) maximum number of motifs to identify =10.

### Chromosomal locations and gene duplication of MADS-box genes

All MADS-box genes of *B. rapa* were BLAST searched (http://www.ncbi.nlm.nih.gov/BLAST/) against each other to identify duplicate genes, with the criteria that both the similarity and query coverage percentage of the candidate genes were > 80% [77]. Positional information for all candidate MADS-box genes along the 10 chromosomes of *B. rapa* were obtained from the *Brassica* database (http://brassicadb.org/brad/) [37]. The map of all genes along the 10 chromosomes and duplication lines among genes were drawn manually.

### Analysis of syntenic relationships

To identify Arabidopsis orthologues of MADS-box genes in *B. rapa,* each candidate MADS-box gene nucleotide sequence was employed in a BLASTX search of the NCBI database (http://blast.ncbi.nlm.nih.gov/Blast.cgi) using *A. thaliana* as reference organism and the best hit *A. thaliana* homologue was considered to be the orthologue of the *B. rapa* MADS-box gene.

### Collection and preparation of plant material

*B. rapa* ‘SUN-3061’ plants were grown in the Department of Horticulture, Sunchon National University, Korea. For the organ study, fresh roots, stems, leaves and flower buds were harvested, frozen immediately in liquid nitrogen, and stored at −80°C for RNA isolation. For the three abiotic stress treatments, two inbred lines of *B. rapa* ssp. *pekinensis* ‘Chiifu’ and ‘Kenshin’ were used. Chiifu originated in temperate regions, whereas Kenshin originated in subtropical and tropical regions [78]. Plants were cultivated under aseptic conditions in semisolid media for 10 d, after which plants were transferred into liquid media to minimize stress during the treatment time. Three stress treatments, cold, drought and salt, were administered over 8 time periods (0 h, 30 min, 1 h, 4 h, 8 h, 12 h, 24 h and 48 h). Plant samples were transferred to the incubator at 4°C to induce cold stress. Drought/desiccation stress was simulated by drying the plants on Whatmann 3 mm filter sheets. To induce salt stress, plant samples were transferred to rectangular petri dishes (72 × 72 × 100 mm) with medium containing 200 mM NaCl for the designed time courses [35]. In each stress experiment, leaves of treated samples were collected and processed to study the expression of different MADS-box genes.

### Microarray expression analysis

Br135K microarray (Brapa_V3_microarray, 3’-Tiling microarray) is a high-density DNA array prepared with Maskless Array Synthesizer (MAS) technology by NimbleGen (http://www.nimblegen.com/). Probes are designed from 41,173 genes of *B. rapa* accession Chiifu-401-42, a Chinese cabbage [36]. For the microarray experiment four-week-old *B. rapa* inbred lines, Chiifu and Kenshin, were treated with cold or freezing stress (4°C, 0°C, −2°C and −4°C). Stress treatments were applied for 2 h and immediately after stress, total and polysomal RNA was extracted from the leaf tissues using the RNeasy Mini kit (Qiagen, USA). RNA protect reagent (Qiagen) and DNA was removed by on-column DNase digestion with the RNase-Free DNase set (Qiagen). Labeling was performed by NimbleGen Systems Inc. (Madison, WI USA), following their standard operating protocol (www.nimblegen.com). The raw data (pair file) was subjected to RMA (Robust Multi-Array Analysis) [79], quantile normalization [80], and background correction as implemented in the NimbleScan software package, version 2.4.27 (Roche NimbleGen, Inc.). To assess the reproducibility of the microarray analysis, we repeated the experiment three times with independently prepared total RNA. The complete microarray data have been deposited in Omics database of NABIC (http://nabic.rda.go.kr) as enrolled number, NC-0024-000001 − NC-0024-000012.

### RT-PCR expression analysis

RT-PCR was conducted using an AMV one step RT-PCR kit (Takara, Japan). Specific primers for all genes were used in RT-PCR, and *Actin* primers for *B. rapa* (FJ969844) were used as a control (Additional file 3: Table S4). PCR was conducted using 50 ng cDNA from the plant and flower organs as templates in master mixes composed of 20 pmol each primer, 150 μM each dNTP, 1.2 U Taq polymerase, 1x Taq polymerase buffer and double-distilled H_2_O diluted to a total volume of 20 μL in 0.5-mL PCR tubes. The samples were subjected to the following conditions: pre-denaturing at 94°C for 5 min, followed by 30 cycles of denaturation at 94°C for 30 s, annealing at 55°C for 30 s and extension at 72°C for 45 s, with a final extension for 5 min at 72°C.

### qPCR expression analysis

Real-time quantitative PCR was performed using 1 μL cDNA in a 20-μL reaction volume employing iTaqTM SYBR® Green Super-mix with ROX (California, USA). The specific primers used for real-time PCR are listed in Additional file 4: Table S5. The conditions for real-time PCR were as follows: 10 min at 95°C, followed by 40 cycles at 95°C for 20 s, 58°C for 20 s, and 72°C for 25 s. The fluorescence was measured following the last step of each cycle, and three replicates were used for each sample. Amplification detection and data analysis were conducted using LightCycler96 (Roche, Germany).

## Additional files

Additional file 1: Table S1. Total number of MADS-box genes within each group of *Arabidopsis*, Rice, Soybean, Maize, Sorghum and *B. rapa.* **Table S2.** Homology analysis of 167 MADS-box genes in *B. rapa.* **Table S3.** Synteny table showing *A. thaliana* orthologous MADS-box gene pairs in *B. rapa*. Additional file 2: Figure S1.
**(a)** Phylogenetic analysis of 138 type I MADS-box proteins from *B.rapa* (67), Arabidopsis (43) and Rice (28). **Figure S1. (b)** Phylogenetic analysis of type II *B. rapa, R*ice and Arabidopsis MADS-box proteins.181 type II MADS-box proteins from *B. rapa* (100), Arabidopsis (43) and rice (38) showing 13 MIKC^c^ clades and MIKC^*^ group as marked in the figure. **FigureS2.** Exon–intron structures of *B.rapa* MADS-box genes. Green boxes, exons; lines, introns. Five groups MIKC^c^, MIKC^*^, Mα, Mβ and Mγ are labeled under type II and type I. Size of each gene can be estimated using the scale (in Kilobase; Kb) on the top of the figure. **Figure S3.** Distribution of Conserved motifs in *Brassica rapa* MADS-box type I proteins identified using MEME search tool. Schematic representation of motifs identified in *B.rapa* MADS-box type I proteins using MEME motif search tool for each group (Mα, Mβ and Mγ) given separately. Different motifs are indicated by different colors, and the names of all members are shown on the left side of the figure. The order of the motifs corresponds to the position of the motifs in individual protein sequences. **Figure S4.** Microarray expression analysis of MADS-box genes in *B. rapa* under different temperature treatment. Here C and K indicates Chiifu and Kenshin, were treated under five (5) temperatures as control (C1&K1), 4°c (C2&K2), 0°c (C3&K3), -2°c (C4&K4), and-4°c (C5&K5). Color bar at the top representing differential expression like purple representing medium level expression where pink to white showing low to no expression. Additional file 3: Table S4. RT-PCR primer list of BrMADSs. Additional file 4: Table S5. Primers for qantitative PCR of BrMADSs.

## Abbreviations

TF: Transcription Factor
BRAD: Brassica database
ORF: Open Reading Frame
MEME: Multiple Em for Motif Elicitation
WGD: Whole Genome Duplication
GSDS: Gene Structure Display Server
MAS: Maskless Array Synthesizer
RMA: Robust Multi-Array Analysis
NRF: National Research Foundation of Korea
