# Supplementary material for: Genome-wide identification and characterization of MADS-box family genes related to organ development and stress resistance in Brassica rapa
Source: BMC Genomics. 2015 Mar 14;16(1):178. doi: 10.1186/s12864-015-1349-z (PMC4422603; doi:10.1186/s12864-015-1349-z)
Supplement: Additional file 1: Table S1. — Total number of MADS-box genes within each group of Arabidopsis, Rice, Soybean, Maize, Sorghum and B. rapa. Table S2. Homology analysis of 167 MADS-box genes in B. rapa. Table S3. Synteny table showing A. thaliana orthologous MADS-box gene pairs in B. rapa. [file 12864_2015_1349_MOESM1_ESM.pdf]

Additional file 1: Table S1

Total number of MADS-box genes within each group of *Arabidopsis*, Rice, Soybean, Maize, Sorghum and *B. rapa*

Sl.	Crop	MIKC[c]	MIKC*	Mα	Mβ	Mγ	Total
1	Arabidopsis[a]	39	6	25	20	16	**107**
2	Rice[b]	38	5	13	9	10	**75**
3	Soybean[c]	67	5	18	5	11	**106**
4	Maize[d]	39	4	27	3	2	**75**
5	Sorghum[d]	33	2	26	2	2	**65**
6	*B. rapa*	89	11	29	16	22	**167**

[a] Parenicova et al. (2003)

[b]Arora et al. (2007)

[c] Shu et al. (20013)

[d] Zhao et al. (2010)

Reference list of additional file 1: table S1

Arora R, Agarwal P, Ray S, Singh AK, Singh VP, Tyagi AK, Kapoor S: **MADS-box gene family in rice: genome-wide identification, organization and expression profiling during reproductive development and stress.** *BMC Genomics* 2007, **8(1):**242.

Parenicova L, de Folter S, Kieffer M, Horner DS, Favalli C, Busscher J, Cook *et al.*: **Molecular and phylogenetic analyses of the complete MADS-box transcription factor family in Arabidopsis.** *Plant Cell* 2003, **15(7):**1538–1551.

Shu Y, Yu D, Wang D, Guo D, Guo C: **Genome-wide survey and expression analysis of the MADS-box gene family in soybean.** *Molecular biology reports* 2013, **40(6):**3901-3911.

Zhao Y, Li X, Chen W, Peng X, Cheng X, Zhu S, Cheng B: **Whole-genome survey and characterization of MADS-box gene family in maize and sorghum.** *Plant Cell Tissue Organ Cult* 2010, 105(2):159–173.

Additional file 1: Table S2
Homology analysis of 167 MADS-box genes in *B. rapa*

Sl.	Gene name	Top matched clones	Name of protein	% Identity	E-value	Top homologous species	References
1	*BrMADS1*	NP182170	inner membrane OXA1-like protein	78	5E-98	*A. thaliana*	(Lin et al. 1999)
2	*BrMADS2*	NP191298	protein agamous-like 18	72	3E-117	*A. thaliana*	(Salanoubat et al. 2000)
3	*BrMADS3*	NP191298	protein agamous-like 18	80	9E-133	*A. thaliana*	(Salanoubat et al. 2000)
4	*BrMADS4*	NP191298	protein agamous-like 18	55	6E-66	*A. thaliana*	(Salanoubat et al. 2000)
5	*BrMADS5*	AAB03807	AGL15 type 2	93	1E-166	*B. napus*	(Heck et al. 1995)
6	*BrMADS6*	ABD77425	agamous-like 15	99	0.0	*B. napus*	Unpublished
7	*BrMADS7*	XP002864937	MADS-box protein AGL27-I	83	1E-80	*A. lyrata subsp. lyrata*	Unpublished
8	*BrMADS8*	NP201311	K-box region and MADS-box transcription factor family protein	71	2E-92	*A. thaliana*	Unpublished
9	*BrMADS9*	NP001078798	agamous-like MADS-box protein AGL31	64	3E-62	*A. thaliana*	Unpublished
10	*BrMADS10*	NP201312	K-box region and MADS-box transcription factor family protein	72	1E-90	*A. thaliana*	Unpublished
11	*BrMADS11*	NP201312	K-box region and MADS-box transcription factor family protein	75	4E-96	*A. thaliana*	Unpublished
12	*BrMADS12*	ABO40820	FLC	99	2E-135	*B. rapa subsp. chinensis*	Unpublished
13	*BrMADS13*	AAK70215	MADS-box protein	100	1E-134	*B. napus*	(Tadege et al. 2001)
14	*BrMADS14*	ADA70732	flowering locus C3	99	2E-134	*B. rapa var. purpuraria*	Unpublished
15	*BrMADS15*	AAK70219	MADS-box protein	73	1E-85	*B. napus*	(Tadege et al. 2001)
16	*BrMADS16*	NP179848	agamous-like MADS-box protein AGL17	85	1E-135	*A. thaliana*	(Lin et al. 1999)
17	*BrMADS17*	NP179848	agamous-like MADS-box protein AGL17	82%	9E-130	*A. thaliana*	(Lin et al. 1999)
18	*BrMADS18*	NP195507	agamous-like MADS-box protein AGL21	91%	2E-152	*A. thaliana*	(Mayer et al. 1999)
19	*BrMADS19*	NP195507	agamous-like MADS-box protein AGL21	86%	6E-140	*A. thaliana*	(Mayer et al. 1999)
20	*BrMADS20*	NP191282	protein agamous-like 16	86%	2E-143	*A. thaliana*	(Salanoubat et al. 2000)
21	*BrMADS21*	NP001078305	protein agamous-like 16	69%	5E-124	*A. thaliana*	(Salanoubat et al. 2000)
22	*BrMADS22*	ABI96182	short vegetative phase protein	100%	2E-172	*B. rapa subsp. Oleifera*	Unpublished
23	*BrMADS23*	ABG24233	short vegetative phase	100%	4E-168	*B. rapa subsp.*	(Lee et al. 2007)

			protein			*Oleifera*	
24	*BrMADS24*	AFM77897	MADS-box protein AGL24	95%	3E-146	*B. napus*	Unpublished
25	*BrMADS25*	NP194184	O-fucosyltransferase family protein	90%	0.0	*A. thaliana*	(Mayer et al. 1999)
26	*BrMADS26*	AEF32148	MADS-box DNA-binding domain transcription factor	100%	6E-179	*B. napus*	(Deng et al. 2012)
27	*BrMADS27*	ADV03948	MADS DNA domain binding transcription factor BnaA	98%	2E-158	*B. napus*	Unpublished
28	*BrMADS28*	ABY59773	MADS-box DNA-binding domain transcription factor	99%	4E-174	*B. napus*	Unpublished
29	*BrMADS29*	ABW74343	PISTILLATA-1	95%	9E-141	*B. napus*	(Deng et al. 2011)
30	*BrMADS30*	AAY63867	PISTILLATA	96%	3E-141	*B. juncea*	Unpublished
31	*BrMADS31*	ABW74343	PISTILLATA-1	95%	9E-94	*B. napus*	(Deng et al. 2011)
32	*BrMADS32*	AAF28894	APETALA3, floral homeotic protein APETALA3 [Brassica rapa subsp. chinensis], APETALA3-4 [Brassica napus]	99%	6E-164	*B.napus*	(Pylatuik et al. 2003)
33	*BrMADS33*	AAB08877	homeotic protein boi1AP3	100%	4E-171	*B. oleracea var. italica*	(Carr and Irish 1997)
34	*BrMADS34*	NP565022	agamous-like MADS-box protein AGL12	93%	2E-133	*A. thaliana*	(Theologis et al. 2000)
35	*BrMADS35*	NP565022	agamous-like MADS-box protein AGL12	91%	2E-131	*A. thaliana*	(Theologis et al. 2000)
36	*BrMADS36*	AFM77891	MADS-box protein AGL20/SOC1	93%	7E-143	*B. napus*	Unpublished
37	*BrMADS37*	AFM77893	MADS-box protein AGL20/SOC1	99%	1E-152	*B. juncea*	Unpublished
38	*BrMADS38*	AFM77891	MADS-box protein AGL20/SOC1	99%	9E-151	*B. napus*	Unpublished
39	*BrMADS39*	NP192925	agamous-like MADS-box protein AGL14	65%	2E-74	*A. thaliana*	(Mayer et al. 1999)
40	*BrMADS40*	NP194026	agamous-like MADS-box protein AGL19	55%	2E-60	*A. thaliana*	(Mayer et al. 1999)
41	*BrMADS41*	NP194025	protein kinase family protein	79%	0.0	*A. thaliana*	(Mayer et al. 1999)
42	*BrMADS42*	NP194026	agamous-like MADS-box protein AGL19	86%	1E-124	*A. thaliana*	(Mayer et al. 1999)
43	*BrMADS43*	NP568952	protein agamous-like 42	80%	1E-114	*A. thaliana*	Unpublished
44	*BrMADS44*	NP568952	protein agamous-like 42	87%	3E-127	*A. thaliana*	Unpublished
45	*BrMADS45*	NP200000	protein agamous-like 71	56%	7E-61	*A. thaliana*	Unpublished
46	*BrMADS46*	NP001078745	MADS-box protein	57%	6E-64	*A. thaliana*	Unpublished
47	*BrMADS47*	NP001078745	MADS-box protein	43%	2E-38	*A. thaliana*	Unpublished
48	*BrMADS48*	XP002865878	MADS-box protein	57%	3E-66	*A. lyrata subsp. lyrata*	Unpublished
49	*BrMADS49*	NP001078745	MADS-box protein	84%	9E-115	*A. thaliana*	Unpublished
50	*BrMADS50*	XP002865878	MADS-box protein	87%	2E-121	*A. lyrata subsp. lyrata*	Unpublished
51	*BrMADS51*	NP001190517	protein agamous-like 71	74%	1E-106	*A. thaliana*	Unpublished
52	*BrMADS52*	NP001190517	protein agamous-like 71	70%	3E-98	*A. thaliana*	Unpublished
53	*BrMADS53*	ACD76823	SEEDSTICK-like	91%	4E-138	*Capsella bursa-*	Unpublished

			protein			*pastoris*	
54	*BrMADS54*	NP192734	agamous-like MADS-box protein AGL11	87%	7E-135	*A. thaliana*	(Mayer et al. 1999)
55	*BrMADS55*	Q01540	RecName: Full=Floral homeotic protein AGAMOUS	100%	0.0	*B. napus*	(Mandel et al. 1992)
56	*BrMADS56*	P17839	RecName: Full=Floral homeotic protein AGAMOUS	92%	3E-167	*A. thaliana*	(Yanofsky et al. 1990)
57	*BrMADS57*	NP191437	agamous-like MADS-box protein AGL1	92%	7E-168	*A. thaliana*	(Salanoubat et al. 2000)
58	*BrMADS58*	NP191437	agamous-like MADS-box protein AGL1	90%	7E-160	*A. thaliana*	(Salanoubat et al. 2000)
59	*BrMADS59*	NP191437	agamous-like MADS-box protein AGL1	92%	1E-163	*A. thaliana*	(Salanoubat et al. 2000)
60	*BrMADS60*	NP565986	agamous-like MADS-box protein AGL5	92%	1E-161	*A. thaliana*	(Lin et al. 1999)
61	*BrMADS61*	Q41276	Full=Floral homeotic protein APETALA 1; AltName: Full=MADS C	95%	7E-168	*Sinapis alba*	(Menzel et al. 1995)
62	*BrMADS62*	Q8GTF4	RecName: Full=Floral homeotic protein APETALA 1 C, Full=Agamous-like MADS-box protein AP1-C	99%	1E-119	*B. oleracea var. botrytis*	(Carr and Irish 1997)
63	*BrMADS63*	Q8GTF5	RecName: Full=Floral homeotic protein APETALA 1 A, Full=Agamous-like MADS-box protein AP1-A	96%	2E-164	*B. oleracea var. botrytis*	(Duclos and Björkman 2008)
64	*BrMADS64*	CAD47852	MADS-box protein FUL-d	98%	2E-169	*B. oleracea var. botrytis*	Unpublished
65	*BrMADS65*	CAD47849	MADS-box protein FUL-a	97%	4E-167	*B. oleracea var. botrytis*	Unpublished
66	*BrMADS66*	Q41274	RecName: Full=Agamous-like MADS-box protein AGL8 homolog	95%	1E-165	*Sinapis alba*	(Menzel et al. 1996)
67	*BrMADS67*	NP189645	protein agamous-like 79	82%	9E-124	*A. thaliana*	(Salanoubat et al. 2000)
68	*BrMADS68*	BAB02228	MADS box transcription factor-like protein	88%	4E-93	*A. thaliana*	(Kaneko et al. 2000)
69	*BrMADS69*	NP189645	protein agamous-like 79	83%	6E-97	*A. thaliana*	(Salanoubat et al. 2000)
70	*BrMADS70*	Q6R4S3	RecName: Full=Transcription factor, AltName: Full=Agamous-like MADS-box protein CAL	100%	0.0	*B. rapa subsp. rapa*	(Purugganan et al. 2000)
71	*BrMADS71*	NP191671	agamous-like MADS-box protein AGL13	67%	6E-101	*A. thaliana*	(Salanoubat et al. 2000)

72	*BrMADS72*	CAD48306	MADS-box protein AGL6-a	89%	3E-157	*B. oleracea var. botrytis*	Unpublished
73	*BrMADS73*	CAD48306	MADS-box protein AGL6-a	83%	1E-151	*B. oleracea var. botrytis*	Unpublished
74	*BrMADS74*	XP002884314	SEPALLATA2	95%	7E-173	*A. lyrata subsp. lyrata*	Unpublished
75	*BrMADS75*	XP002884314	SEPALLATA2	92%	6E-171	*A. lyrata subsp. lyrata*	Unpublished
76	*BrMADS76*	O04067	RecName: Full=Agamous-like MADS-box protein AGL9 homolog	88%	1E-151	*Sinapis alba*	(Bonhomme et al. 1997)
77	*BrMADS77*	O04067	RecName: Full=Agamous-like MADS-box protein AGL9 homolog	93%	1E-169	*Sinapis alba*	(Bonhomme et al. 1997)
78	*BrMADS78*	CAD48302	MADS-box protein AGL3-a	83%	1E-162	*B. oleracea var. botrytis*	Unpublished
79	*BrMADS79*	CAD48302	MADS-box protein AGL3-a	85%	7E-148	*B. oleracea var. botrytis*	Unpublished
80	*BrMADS80*	CAD48302	MADS-box protein AGL3-a	97%	0.0	*B. oleracea var. botrytis*	Unpublished
81	*BrMADS81*	O04067	RecName: Full=Agamous-like MADS-box protein AGL9 homolog	96%	6E-178	*Sinapis alba*	(Bonhomme et al. 1997)
82	*BrMADS82*	AAA32732	transcription factor, SEPALLATA1	93%	2E-168	*A. thaliana*	(Ma et al. 1991)
83	*BrMADS83*	CAD48303	MADS-box protein SEP1-a	100%	0.0	*B. oleracea var. botrytis*	Unpublished
84	*BrMADS84*	BAD93735	MADS transcription factor-like protein	95%	7E-32	*A. thaliana*	Unpublished
85	*BrMADS85*	NP974450	protein agamous-like 18	75%	8E-85	*A. thaliana*	(Salanoubat et al. 2000)
86	*BrMADS86*	BAD93735.1	MADS transcription factor-like protein	76%	2E-20	*A. thaliana*	Unpublished
87	*BrMADS87*	NP974450	protein agamous-like 18	39%	2E-07	*A. thaliana*	(Salanoubat et al. 2000)
88	*BrMADS88*	NP565022	agamous-like MADS-box protein AGL12	98%	2E-34	*A. thaliana*	(Theologis et al. 2000)
89	*BrMADS89*	ABI20790	MADS-box protein	47%	1E-25	*A. lyrata subsp. lyrata]*	Unpublished
90	*BrMADS90*	ADU56838	MADS-box protein Md subfamily	37%	5E-28	*Coffea arabica*	Unpublished
91	*BrMADS91*	NP177918	protein agamous-like 67	85%	1E-107	*A. thaliana*	(Theologis et al. 2000)
92	*BrMADS92*	NP173632	protein AGAMOUS-like 104	85%	0.0	*A. thaliana*	(Theologis et al. 2000)
93	*BrMADS93*	NP173632	protein AGAMOUS-like 104	78%	1E-173	*A. thaliana*	Theologis A et al (2000)
94	*BrMADS94*	NP177921	protein agamous-like 66	84%	0.0	*A. thaliana*	(Theologis et al. 2000)
95	*BrMADS95*	NP173310	protein AGAMOUS-like 65	78%	0.0	*A. thaliana*	(Theologis et al. 2000)
96	*BrMADS96*	BAC99091	MADS-box protein	73%	3E-110	*A. thaliana*	(Kofuji et al. 2003)
97	*BrMADS97*	NP173310	protein AGAMOUS-like 65	86%	0.0	*A. thaliana*	(Theologis et al. 2000)
98	*BrMADS98*	NP001077873	protein agamous-like 30	81%	0.0	*A. thaliana*	(Lin et al. 1999)

99	*BrMADS99*	NP001077873	protein agamous-like 30	78%	0.0	*A. thaliana*	(Lin et al. 1999)
100	*BrMADS100*	NP177113	protein agamous-like 94	72%	1E-173	*A. thaliana*	(Theologis et al. 2000)
101	*BrMADS101*	XP002882329	MADS-box family protein	54%	2E-48	*A. lyrata subsp. lyrata*	Unpublished
102	*BrMADS102*	XP002882329	MADS-box family protein	50%	8E-44	*A. lyrata subsp. lyrata*	Unpublished
103	*BrMADS103*	XP002882329	MADS-box family protein	53%	1E-51	*A. lyrata subsp. lyrata*	Unpublished
104	*BrMADS104*	NP001077625	protein agamous-like 64	49%	1E-47	*A. thaliana*	(Theologis et al. 2000)
105	*BrMADS105*	XP002883198	hypothetical protein ARALYDRAFT_898356	61%	1E-96	*A. lyrata subsp. lyrata*	Unpublished
106	*BrMADS106*	NP200852	agamous-like MADS-box protein AGL62	55%	2E-100	*A. thaliana*	Unpublished
107	*BrMADS107*	XP002864686	MADS-box protein	44%	1E-65	*A. lyrata subsp. lyrata*	Unpublished
108	*BrMADS108*	XP002864686	MADS-box protein	61%	3E-97	*A. lyrata subsp. lyrata*	Unpublished
109	*BrMADS109*	NP195377	MADS-box transcription factor family protein	44%	8E-45	*A. thaliana*	(Mayer et al. 1999)
110	*BrMADS110*	NP200852	agamous-like MADS-box protein AGL62	45%	1E-56	*A. thaliana*	Unpublished
111	*BrMADS111*	NP850058	agamous-like MADS-box protein AGL61	76%	1E-82	*A. thaliana*	(Lin et al. 1999)
112	*BrMADS112*	NP850058	agamous-like MADS-box protein AGL61	74%	1E-104	*A. thaliana*	(Lin et al. 1999)
113	*BrMADS113*	NP850058	agamous-like MADS-box protein AGL61	58%	1E-54	*A. thaliana*	(Lin et al. 1999)
114	*BrMADS114*	NP187320	protein agamous-like 91	69%	3E-71	*A. thaliana*	(Salanoubat et al. 2000)
115	*BrMADS115*	NP180991	protein agamous-like 29	76%	9E-90	*A. thaliana*	(Lin et al. 1999)
116	*BrMADS116*	NP177379	MADS-box protein	68%	2E-84	*A. thaliana*	(Theologis et al. 2000)
117	*BrMADS117*	XP002892945	MADS-box protein	69%	8E-86	*A. lyrata subsp. lyrata*	Unpublished
118	*BrMADS118*	NP198678	MADS-box protein AGL73	53%	1E-48	*A. thaliana*	Unpublished
119	*BrMADS119*	NP187857	MADS-box family protein	35%	3E-08	*A. thaliana*	(Salanoubat et al. 2000)
120	*BrMADS120*	NP198678	MADS-box protein AGL73	41%	9E-66	*A. thaliana*	Unpublished
121	*BrMADS121*	NP198678	MADS-box protein AGL73	46%	1E-62	*A. thaliana*	Unpublished
122	*BrMADS122*	NP199753	MADS-box transcription factor family protein	45%	5E-52	*A. thaliana*	Unpublished
123	*BrMADS123*	NP199753	MADS-box transcription factor family protein	56%	2E-58	*A. thaliana*	Unpublished
124	*BrMADS124*	NP176288	protein agamous-like 55	69%	3E-75	*A. thaliana*	(Theologis et al. 2000)
125	*BrMADS125*	NP176288	protein agamous-like 55	64%	2E-76	*A. thaliana*	Unpublished
126	*BrMADS126*	NP196084	protein agamous-like 99	39%	9E-50	*A. thaliana*	Unpublished
127	*BrMADS127*	NP187857	MADS-box family protein	48%	8E-26	*A. thaliana*	Unpublished
128	*BrMADS128*	NP175249	MADS-box domain-containing protein	49%	2E-73	*A. thaliana*	(Theologis et al. 2000)

129	*BrMADS129*	XP002864686	MADS-box protein	55%	1E-82	*A. lyrata subsp. lyrata*	Unpublished
130	*BrMADS130*	NP200380	MADS-box protein AGL47	70%	1E-138	*A. thaliana*	Unpublished
131	*BrMADS131*	XP002864598	MADS-box family protein	61%	4E-118	*A. lyrata subsp. lyrata*	Unpublished
132	*BrMADS132*	NP188495	protein agamous-like 103	47%	4E-69	*A. thaliana*	(Salanoubat et al. 2000)
133	*BrMADS133*	NP188495	protein agamous-like 103	58%	1E-106	*A. thaliana*	(Salanoubat et al. 2000)
134	*BrMADS134*	NP201336	protein agamous-like 78	51%	5E-90	*A. thaliana*	Unpublished
135	*BrMADS135*	NP198936	protein agamous-like 75	43%	8E-50	*A. thaliana*	Unpublished
136	*BrMADS136*	NP201336	protein agamous-like 78	45%	3E-54	*A. thaliana*	Unpublished
137	*BrMADS137*	NP198936	protein agamous-like 75	49%	2E-66	*A. thaliana*	Unpublished
138	*BrMADS138*	XP002872250	agamous-like MADS-box protein AGL93	37%	1e-32	*A. lyrata subsp. lyrata*	Unpublished
139	*BrMADS139*	XP002872250	MADS-box family protein	39%	4E-36	*A. lyrata subsp. lyrata*	Unpublished
140	*BrMADS140*	NP198061	protein agamous-like 54	43%	2E-30	*A. thaliana*	Unpublished
141	*BrMADS141*	XP002874408	MADS-box family protein	39%	3E-39	*A. lyrata subsp. lyrata*	Unpublished
142	*BrMADS142*	XP002872250	MADS-box family protein	38%	2E-31	*A. lyrata subsp. lyrata*	Unpublished
143	*BrMADS143*	XP002872250	MADS-box family protein	44%	1E-48	*A. lyrata subsp. lyrata*	Unpublished
144	*BrMADS144*	NP198059	protein agamous-like 53	41%	3E-40	*A. thaliana*	Unpublished
145	*BrMADS145*	NP198059	protein agamous-like 53	39%	1E-39	*A. thaliana*	Unpublished
146	*BrMADS146*	NP180438	protein agamous-like 46	49%	2E-72	*A. thaliana*	(Lin et al. 1999)
147	*BrMADS147*	NP180438	protein agamous-like 46	50%	5E-38	*A. thaliana*	(Lin et al. 1999)
148	*BrMADS148*	NP180438	protein agamous-like 46	41%	1E-67	*A. thaliana*	(Lin et al. 1999)
149	*BrMADS149*	NP180438	protein agamous-like 46	41%	8E-68	*A. thaliana*	(Lin et al. 1999)
150	*BrMADS150*	NP187237	MADS-box domain-containing protein	53%	4E-96	*A. thaliana*	(Salanoubat et al. 2000)
151	*BrMADS151*	NP850882	MADS-box protein AGL35	61%	4E-81	*A. thaliana*	Unpublished
152	*BrMADS152*	NP850882	MADS-box protein AGL35	55%	6E-57	*A. thaliana*	Unpublished
153	*BrMADS153*	NP199678	agamous-like MADS-box protein AGL80	60%	7E-107	*A. thaliana*	Unpublished
154	*BrMADS154*	NP199678	agamous-like MADS-box protein AGL80	60%	3E-107	*A. thaliana*	Unpublished
155	*BrMADS155*	NP199678	agamous-like MADS-box protein	53%	7E-83	*A. thaliana*	Unpublished
156	*BrMADS156*	NP176712	MADS-box transcription factor PHERES 1AltName: Full=Agamous-like MADS-box protein	36%	9E-38	*A. thaliana*	(Theologis et al. 2000)
157	*BrMADS157*	BAN63704	agamous-like MADS-box protein AGL36, partial.	36%	7E-46	*A. halleri subsp. gemmifera*	Unpublished
158	*BrMADS158*	NP176712	MADS-box transcription factor PHERES 1 AltName: Full=Agamous-like	36%	2E-35	*A. thaliana*	(Theologis et al. 2000)

159	*BrMADS159*	NP850880	MADS-box protein agamous-like MADS-box protein AGL36, partial	33%	8E-33	*A. halleri subsp. halleri*	Unpublished
160	*BrMADS160*	NP174444	agamous-like MADS-box protein AGL86	36%	5E-52	*A. thaliana*	(Theologis et al. 2000)
161	*BrMADS161*	NP973886	protein AGAMOUS-like 87	72%	5E-79	*A. thaliana*	(Theologis et al. 2000)
162	*BrMADS162*	NP973886	protein AGAMOUS-like 87	76%	2E-83	*A. thaliana*	(Theologis et al. 2000)
163	*BrMADS163*	NP196268	protein agamous-like 96	45%	9E-61	*A. thaliana*	Unpublished
164	*BrMADS164*	NP196268	protein agamous-like 96	49%	4E-46	*A. thaliana*	Unpublished
165	*BrMADS165*	NP196268	protein agamous-like 96	48%	1E-39	*A. thaliana*	Unpublished
166	*BrMADS166*	NP196268	protein agamous-like 96	46%	3E-35	*A. thaliana*	Unpublished
167	*BrMADS167*	NP181550	protein agamous-like 48	41%	3E-72	*A. thaliana*	(Lin et al. 1999)

Reference list of additional file 1: table S2

Bonhomme F, Sommer H, Bernier G, Jacqmard A: **Characterization of SaMADS D from Sinapis alba suggests a dual function of the gene: in inflorescence development and floral organogenesis.** *Plant Mol Biol* 1997, **34(4):**573-582.

Carr SM, Irish VF: **Floral homeotic gene expression defines developmental arrest stages in Brassica oleracea L. vars. botrytis and italica.** *Planta* 1997, **201(2):**179-188.

Deng W, Chen G, Peng F, Truksa M, Snyder CL, Weselake RJ: **Transparent testa16 plays multiple roles in plant development and is involved in lipid synthesis and embryo development in canola.** *Plant Physiol* 2012, **160(2):**978-89.

Deng W, Zhou L, Zhou Y, Wang Y, Wang M, Zhao Y: **Isolation and characterization of three duplicated PISTILLATA genes in Brassica napus.** *Mol Biol Rep* 2011, **38(5):**3113-20.

Duclos DV, Björkman T: **Meristem identity gene expression during curd proliferation and flower initiation in *Brassica oleracea*.** *J Exp Bot* 2008, **59(2):**421-33.

Heck GR, Perry SE, Nichols KW, Fernandez DE: **AGL15, a MADS domain protein expressed in developing embryos.** *Plant Cell* 1995, **7(8):**1271-82.

Kaneko T, Katoh T, Sato S, Nakamura A *et al.*: **Structural analysis of Arabidopsis thaliana chromosome 3. II. Sequence features of the 4,251,695 bp regions covered by 90 P1, TAC and BAC clones.** *DNA Res* 2000, **7(3):**217-721.

Kofuji R, Sumikawa N, Yamasaki M, Kondo K: **Evolution and divergence of the MADS-box gene family based on genome-wide expression analyses.** *Mol Biol Evol* 2003, **20(12):**1963-1977.

Lee JH, Park SH, Lee JS, Ahn JH: **A conserved role of SHORT VEGETATIVE PHASE (SVP) in controlling flowering time of Brassica plants.** *Biochim Biophys Acta* 2007, **1769(7-8):**455-461.

Lin X, Kaul S, Rounsley S, Shea TP *et al.*: **Sequence and analysis of chromosome 2 of the plant Arabidopsis thaliana.** Nature 1999, **402(6763):**761-768

Ma H, Yanofsky MF, Meyerowitz EM: **AGL1-AGL6, an Arabidopsis gene family with similarity to floral homeotic and transcription factor genes.** *Genes Dev* 1991, **5(3):**484-495.

Mandel MA, Bowman JL, Kempin SA, Ma H *et al.*: **Manipulation of flower structure in transgenic tobacco.** *Cell* 1992, **71(1):**133-143.

Mayer K, Schüller C, Wambutt R, Murphy G *et al.*: **Sequence and analysis of chromosome 4 of the plant Arabidopsis thaliana.** *Nature* 1999, **402(6763):**769-777.

Menzel G, Apel K, Melzer S: **Isolation and analysis of SaMADS C, the APETALA 1 cDNA homolog from mustard.** *Plant Physiol* 1995, **108(2):**853-854.

Menzel G, Apel K, Melzer S: **Identification of two MADS box genes that are expressed in the apical meristem of the long-day plant Sinapis alba in transition to flowering.** *Plant J* 1996, **9(3):**399-408.

Purugganan MD, Boyles AL, Suddith JI: **Variation and selection at the CAULIFLOWER floral homeotic gene accompanying the evolution of domesticated *Brassica oleracea*.** *Genetics* 2000, **155(2):**855-862.

Pylatuik JD, Lindsay DL, Davis AR, Bonham-Smith PC: **Isolation and characterization of a Brassica napus cDNA corresponding to a B-class floral development gene.** *J Exp Bot* 2003, **54(391):**2385-7.

Salanoubat M, Lemcke K, Rieger M, Ansorge W *et al.*: **European Union Chromosome 3 Arabidopsis Sequencing Consortium; Institute for Genomic Research; Kazusa DNA Research Institute.** *Nature* 2000, **408(6814):**820-822.

Tadege M, Sheldon CC, Helliwell CA, Stoutjesdijk P, Dennis ES, Peacock WJ: **Control of flowering time by FLC orthologues in Brassica napus.** *Plant J* 2001, **28(5):**545-53.

Theologis A, Ecker JR, Palm CJ, Federspiel NA *et al.*: **Sequence and analysis of chromosome 1 of the plant Arabidopsis thaliana.** *Nature* 2000, **408(6814):**816-820.

Yanofsky MF, Ma H, Bowman JL et al.: **The protein encoded by the Arabidopsis homeotic gene agamous resembles transcription factors.** *Nature* 1990, **346(6279):**35-39.

Additional file 1: Table S3

Synteny table showing *A. thaliana* orthologous MADS-box gene pairs in *B. rapa*

Sl. no.	Arabidopsis MADS-box genes	Arabidopsis family/subfamily	Orthologous MADS-box gene in *B. rapa*	Best BLASTX value			Protein BLAST results of *B. rapa* MADS-box genes		
				Query %	Identity %	E-value	Gene name	Identity %	Query %
1.	*AGL18*	AGL15-like	*BrMADS1*	19	73	2e-24	BrMADS86	98	20
			BrMADS2	99	72	5e-116	BrMADS3	80	100
			BrMADS3	99	77	2e-132	BrMADS2	80	100
			BrMADS4	95	55	2e-73	BrMADS2	59	97
			BrMADS84	95	95	1e-38	BrMADS3	97	98
			BrMADS85	98	75	2e-82	BrMADS3	83	99
			BrMADS86	95	76	2e-82	BrMADS1	98	100
2.	*AGL15*	AGL15-like	*BrMADS5*	85	75	7e-124			
			BrMADS6	92	75	4e-129			
			BrMADS87	90	70	1e-46			
3.	*AGL69*	FLC-like	*BrMADS7*	74	65	4e-72			
			BrMADS9	87	64	3e-61			
4.	*AGL27/FLM*	FLC-like	*BrMADS8*	98	73	7e-75			
			BrMADS10	98	68	2e-70			
			BrMADS11	98	68	6e-74			
5.	*AGL25/FLC*	FLC-like	*BrMADS12*	98	85	1e-96	BrMADS14	88	99
			BrMADS13	98	86	5e-97	BrMADS12	87	95
			BrMADS14	99	83	9e-96	BrMADS12	88	99
			BrMADS15	84	88	2e-47	BrMADS14	58	100
6.	*AGL17*	AGL17-like	*BrMADS16*	99	85	4e-143			
			BrMADS17	99	82	3e-137			
7.	*AGL21*	AGL17-like	*BrMADS18*	99	91	5e-160			
			BrMADS19	99	86	2e-147			
8.	*AGL16*	AGL17-like	*BrMADS20*	99	86	2e-143			
			BrMADS21	99	66	4e-115			
9.	*AGL22/SVP*	STMADS11-like	*BrMADS22*	99	92	1e-163			
			BrMADS23	97	91	7e-158			
10.	*AGL24*	STMADS11-like	*BrMADS24*	97	86	3e-113			
			BrMADS25	97	85	9e-108			
11.	*AGL32/TT16*	GGM13-like	*BrMADS26*	73	75	2e-127			
			BrMADS27	80	71	6e-127			
			BrMADS28	77	79	5e-134			
12.	*PISTILLATA*	GLO-like	*BrMADS29*	99	90	9e-131			
			BrMADS30	99	92	1e-132			
			BrMADS31	95	94	9e-101			
13.	*APETALA3*	DEF-like	*BrMADS32*	99	91	2e-161			
			BrMADS33	99	95	3e-171			
14.	*AGL12*	AGL12-like	*BrMADS34*	99	93	1e-140			
			BrMADS35	99	90	4e-131			
			BrMADS88	96	98	1e-40			
15.	*AGL20/SOC1*	TM3-like	*BrMADS36*	99	93	8e-119			
			BrMADS37	99	95	2e-133			
			BrMADS38	99	95	8e-123			
16.	*AGL14*	TM3-like	*BrMADS39*	99	65	2e-78			
17.	*AGL19*	TM3-like	*BrMADS40*	98	55	1e-68			
			BrMADS41	16	91	1e-50			
			BrMADS42	98	86	4e-125			
18.	*AGL42*	TM3-like	*BrMADS43*	99	80	1e-103			
			BrMADS44	99	87	9e-117			

19.	*AGL72*	TM3-like	*BrMADS45*	97	56	2e-68	BrMADS46	75	97
			BrMADS46	96	52	2e-62	BrMADS45	75	96
			BrMADS47	99	40	9e-38	BrMADS46	50	100
			BrMADS48	97	55	3e-70	BrMADS45	67	99
			BrMADS49	99	80	3e-119	BrMADS50	90	95
			BrMADS50	99	82	2e-124	BrMADS49	90	97
			BrMADS89	82	45	4e-27	BrMADS46	60	86
20.	*AGL71*	FLC-like	*BrMADS51*	99	68	7e-94			
			BrMADS52	99	67	2e-94			
21.	*AGL11*	AGAMOUS-like	*BrMADS53*	90	90	7e-144			
			BrMADS54	90	87	9e-142			
22.	*AGAMOUS*	AGAMOUS-like	*BrMADS55*	99	95	2e-161			
			BrMADS56	99	92	1e-160			
23.	*AGL1/SHP1*	AGAMOUS-like	*BrMADS57*	99	92	2e-175			
			BrMADS58	82	90	1e-161			
			BrMADS59	99	92	1e-158			
24.	*AGL5/SHP2*	AGAMOUS-like	*BrMADS60*	99	92	2e-154			
25.	*AGL7/AP1*		*BrMADS61*	99	94	2e-174			
			BrMADS62	66	97	2e-124			
			BrMADS63	99	92	2e-165			
26.	*AGL8/FUL*	SQUA-like	*BrMADS64*	97	92	9e-161			
			BrMADS65	98	94	3e-170			
			BrMADS66	98	92	2e-160			
27.	*AGL79*	SQUA-like	*BrMADS67*	99	82	8e-123			
			BrMADS68	85	88	7e-99			
			BrMADS69	91	82	2e-103			
28.	*AGL10/CAL*	SQUA-like	*BrMADS70*	99	84	4e-157			
29.	*AGL13*	AGL6-like	*BrMADS71*	99	66	2e-102			
30.	*AGL6*	AGL6-like	*BrMADS72*	99	81	3e-136			
			BrMADS73	99	82	3e-143			
31.	*AGL4/SEP2*	AGl2-like	*BrMADS74*	99	94	1e-178			
			BrMADS75	99	92	5e-177			
32.	*AGL9/SEP3*	AGl2-like	*BrMADS76*	97	86	8e-146			
			BrMADS77	99	92	2e-168			
			BrMADS81	99	91	3e-163			
33.	*AGL3/SEP4*	AGl2-like	*BrMADS78*	99	82	7e-156			
			BrMADS79	92	83	2e-143			
			BrMADS80	99	82	5e-157			
34.	*AGL2/SEP1*	AGl2-like	*BrMADS82*	99	90	2e-158			
			BrMADS83	99	89	7e-158			
35.	*AGL67*	MIKC*	*BrMADS90*	91	30	6e-28			
			BrMADS91	53	85	6e-103			
36.	*AGL104*	MIKC*	*BrMADS92*	99	85	0.0			
			BrMADS93	99	78	6e-167			
37.	*AGL66*	MIKC*	*BrMADS94*	99	84	0.0			
38.	*AGL65*	MIKC*	*BrMADS95*	99	78	0.0			
			BrMADS96	91	72	1e-93			
			BrMADS97	99	86	0.0			
39.	*AGL30*	MIKC*	*BrMADS98*	97	80	0.0			
			BrMADS99	97	78	4e-177			
40.	*AGL94*	MIKC*	*BrMADS100*	66	72	2e-161			
41.	*AGL57*	Mα	*BrMADS101*	75	53	3e-42			
			BrMADS103	83	50	7e-44			
42.	*AGL58*	Mα	*BrMADS102*	94	47	2e-38			
43.	*AGL64*	Mα	*BrMADS104*	96	49	2e-48			
44.	*AGL28*	Mα	*BrMADS105*	90	41	2e-44			

45.	*AGL62*	Mα	*BrMADS106*	99	55	8e-102	BrMADS105	66	94
			BrMADS107	98	42	1e-61	BrMADS108	46	97
			BrMADS108	97	57	6e-93	BrMADS106	50	98
			BrMADS113	91	52	2e-50	BrMADS111	61	80
46.	*AGL40*	Mα	*BrMADS109*	68	43	1e-50			
47.	*AGL23*	Mα	*BrMADS110*	93	41	2e-41			
			BrMADS129	85	44	9e-43			
48.	*AGL61*	Mα	*BrMADS111*	61	76	2e-90			
			BrMADS112	79	74	4e-112			
49.	*AGL91*	Mα	*BrMADS114*	98	69	3e-78			
50.	*AGL29*	Mα	*BrMADS115*	98	71	2e-89			
51.	*AGL60*	Mα	*BrMADS116*	88	69	9e-88			
52.	*AGL100*	Mα	*BrMADS117*	96	67	4e-75			
53.	*AGL84*	Mα	*BrMADS118*	60	59	6e-55			
			BrMADS119	49	50	2e-27			
			BrMADS123	75	56	3e-49			
54.	*AGL73*	Mα	*BrMADS120*	96	41	4e-73			
			BrMADS121	78	46	5e-70			
55.	*AGL74*	Mα	*BrMADS122*	57	56	1e-44			
			BrMADS127	77	31	2e-14			
56.	*AGL55*	Mα	*BrMADS124*	89	69	4e-70			
			BrMADS125	98	64	3e-67			
57.	*AGL97*	Mα	*BrMADS126*	78	46	2e-39			
58.	*AGL39*	Mα	*BrMADS128*	88	45	5e-59			
59.	*AGL47*	Mβ	*BrMADS130*	99	70	4e-146			
60.	*AGL82*	Mβ	*BrMADS131*	98	61	2e-116			
61.	*AGL103*	Mβ	*BrMADS132*	86	47	5e-71			
			BrMADS133	86	56	6e-104			
62.	*AGL52*	Mβ	*BrMADS134*	94	49	5e-75			
63.	*AGL76*	Mβ	*BrMADS135*	92	44	4e-53			
			BrMADS136	90	47	3e-58			
			BrMADS137	93	47	1e-63			
64.	*AGL93*	Mβ	*BrMADS138*	90	36	3e-37			
			BrMADS139	77	38	2e-39			
65.	*AGL89*	Mβ	*BrMADS140*	69	45	5e-34	BrMADS139	94	100
			BrMADS141	89	40	4e-44	BrMADS138	74	98
			BrMADS142	62	39	2e-35	BrMADS143	86	94
			BrMADS143	80	41	3e-40	BrMADS142	86	96
			BrMADS144	80	41	1e-38	BrMADS143	65	99
66.	*AGL101*	Mβ	*BrMADS145*	98	46	4e-28			
67.	*AGL46*	Mγ	*BrMADS146*	89	43	2e-57	BrMADS150	52	85
			BrMADS147	99	50	3e-46	BrMADS146	70	100
			BrMADS148	97	38	3e-71	BrMADS149	99	100
			BrMADS149	97	39	2e-71	BrMADS148	99	100
68.	*AGL45*	Mγ	*BrMADS150*	99	53	2e-107			
69.	*AGL35*	Mγ	*BrMADS151*	99	59	1e-77			
			BrMADS152	87	54	9e-55			
70.	*AGL80*	Mγ	*BrMADS153*	72	61	4e-103			
			BrMADS154	72	62	6e-99			
			BrMADS155	72	52	2e-77			
71.	*AGL37*	Mγ	*BrMADS156*	73	39	6e-44			
72.	*AGL36*	Mγ	*BrMADS157*	78	34	4e-43			
73.	*AGL92*	Mγ	*BrMADS158*	73	40	2e-31			
			BrMADS159	78	29	8e-24			
			BrMADS160	83	41	3e-48			
74.	*AGL87*	Mγ	*BrMADS161*	99	72	1e-86			

			BrMADS162	97	76	4e-91			
75.	*AGL96*	Mγ	*BrMADS163*	98	44	2e-68	BrMADS164	46	100
			BrMADS164	85	49	1e-46	BrMADS165	71	98
			BrMADS165	76	48	1e-46	BrMADS164	71	98
			BrMADS166	80	46	2e-36	BrMADS165	64	98
76.	*AGL95*	Mγ	*BrMADS167*	83	52	4e-56			
