# Supplementary material for: Genome-wide identification and characterization of MADS-box family genes related to organ development and stress resistance in Brassica rapa
Source: BMC Genomics. 2015 Mar 14;16(1):178. doi: 10.1186/s12864-015-1349-z (PMC4422603; doi:10.1186/s12864-015-1349-z)
Supplement: Additional file 2: Figure S1. — (a) Phylogenetic analysis of 138 type I MADS-box proteins from B.rapa (67), Arabidopsis (43) and Rice (28). Figure S1. (b) Phylogenetic analysis of type II B. rapa, Rice and Arabidopsis MADS-box proteins.181 type II MADS-box proteins from B. rapa (100), Arabidopsis (43) and rice (38) showing 13 MIKCc clades and MIKC* group as marked in the figure. FigureS2. Exon–intron structures of B.rapa MADS-box genes. Green boxes, exons; lines, introns. Five groups MIKCc, MIKC*, Mα, Mβ and Mγ are labeled under type II and type I. Size of each gene can be estimated using the scale (in Kilobase; Kb) on the top of the figure. Figure S3. Distribution of Conserved motifs in Brassica rapa MADS-box type I proteins identified using MEME search tool. Schematic representation of motifs identified in B.rapa MADS-box type I proteins using MEME motif search tool for each group (Mα, Mβ and Mγ) given separately. Different motifs are indicated by different colors, and the names of all members are shown on the left side of the figure. The order of the motifs corresponds to the position of the motifs in individual protein sequences. Figure S4. Microarray expression analysis of MADS-box genes in B. rapa under different temperature treatment. Here C and K indicates Chiifu and Kenshin, were treated under five (5) temperatures as control (C1&K1), 4°c (C2&K2), 0°c (C3&K3), -2°c (C4&K4), and-4°c (C5&K5). Color bar at the top representing differential expression like purple representing medium level expression where pink to white showing low to no expression. [file 12864_2015_1349_MOESM2_ESM.pdf]

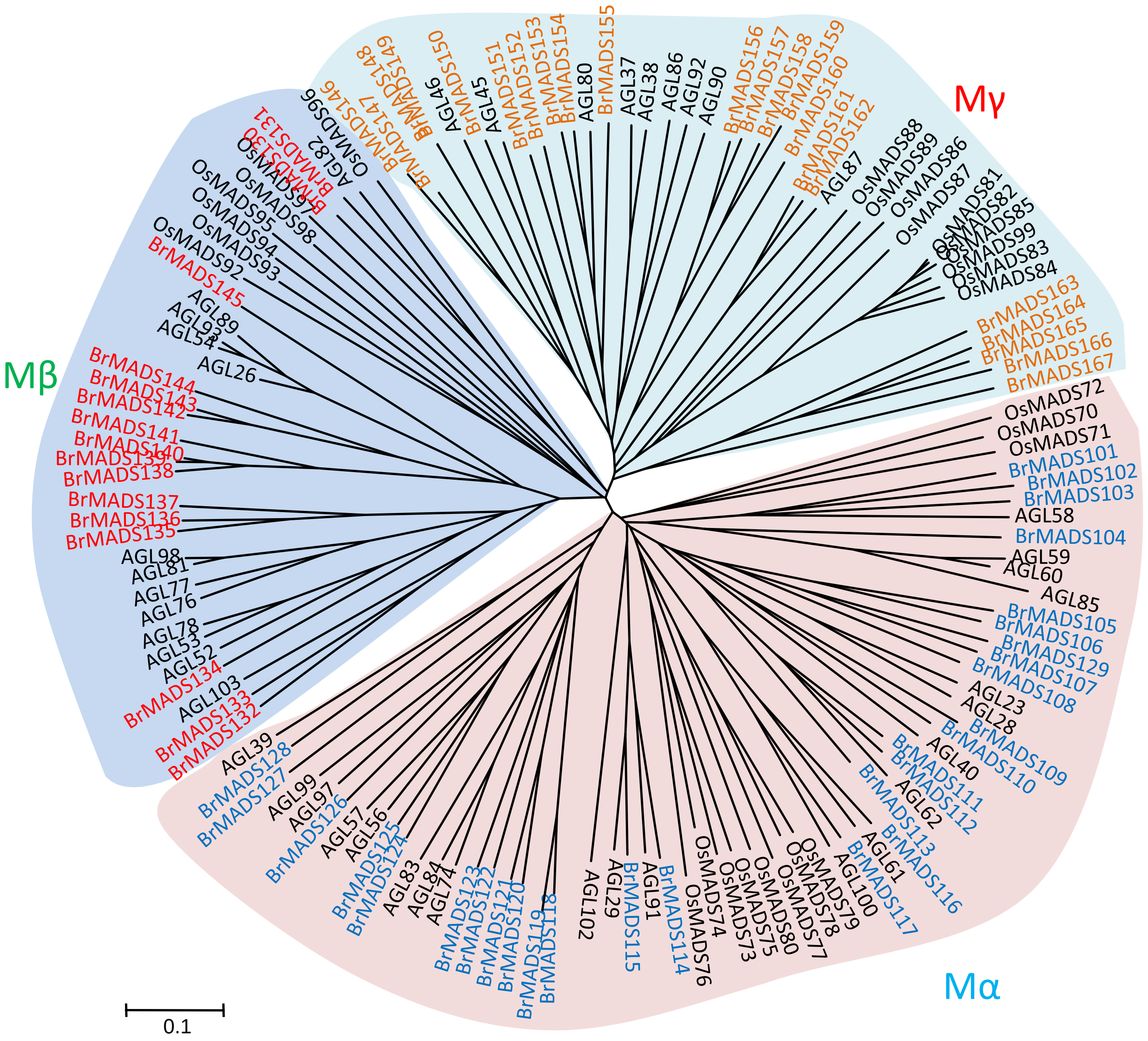


**Additional file 2: Figure S1 (a)**

Phylogenetic analysis of 138 type I MADS-box proteins from *B. rapa* (67), Arabidopsis (43) and Rice (28).

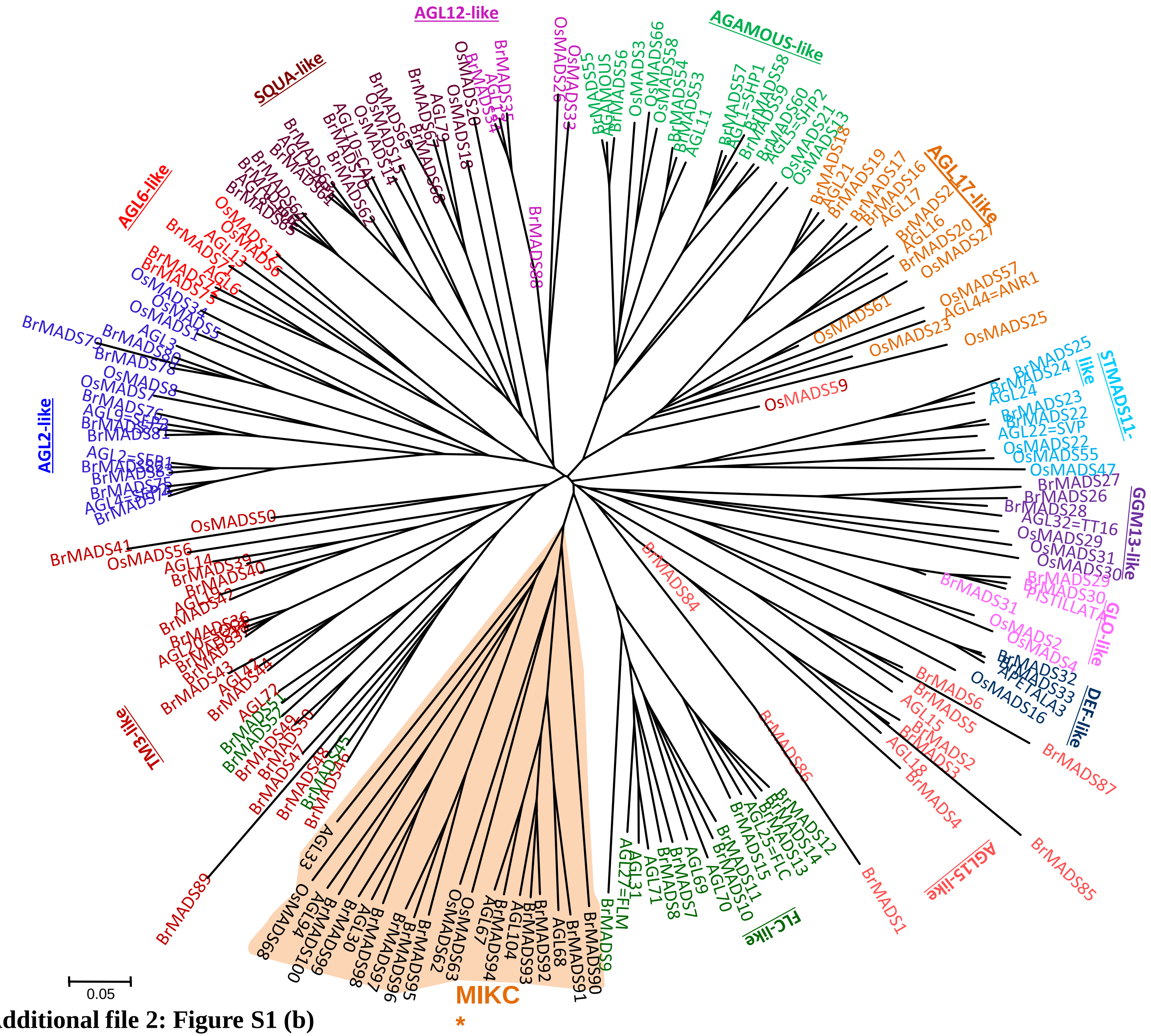


**Additional file 2: Figure S1 (b)**

Phylogenetic analysis of type II *B. rapa, R*ice and Arabidopsis MADS-box proteins. 181 type II MADS-box proteins from *B. rapa* (100), Arabidopsis (43) and rice (38) showing 13 MIKC$^{c}$ clades and MIKC* group as marked in the figure.

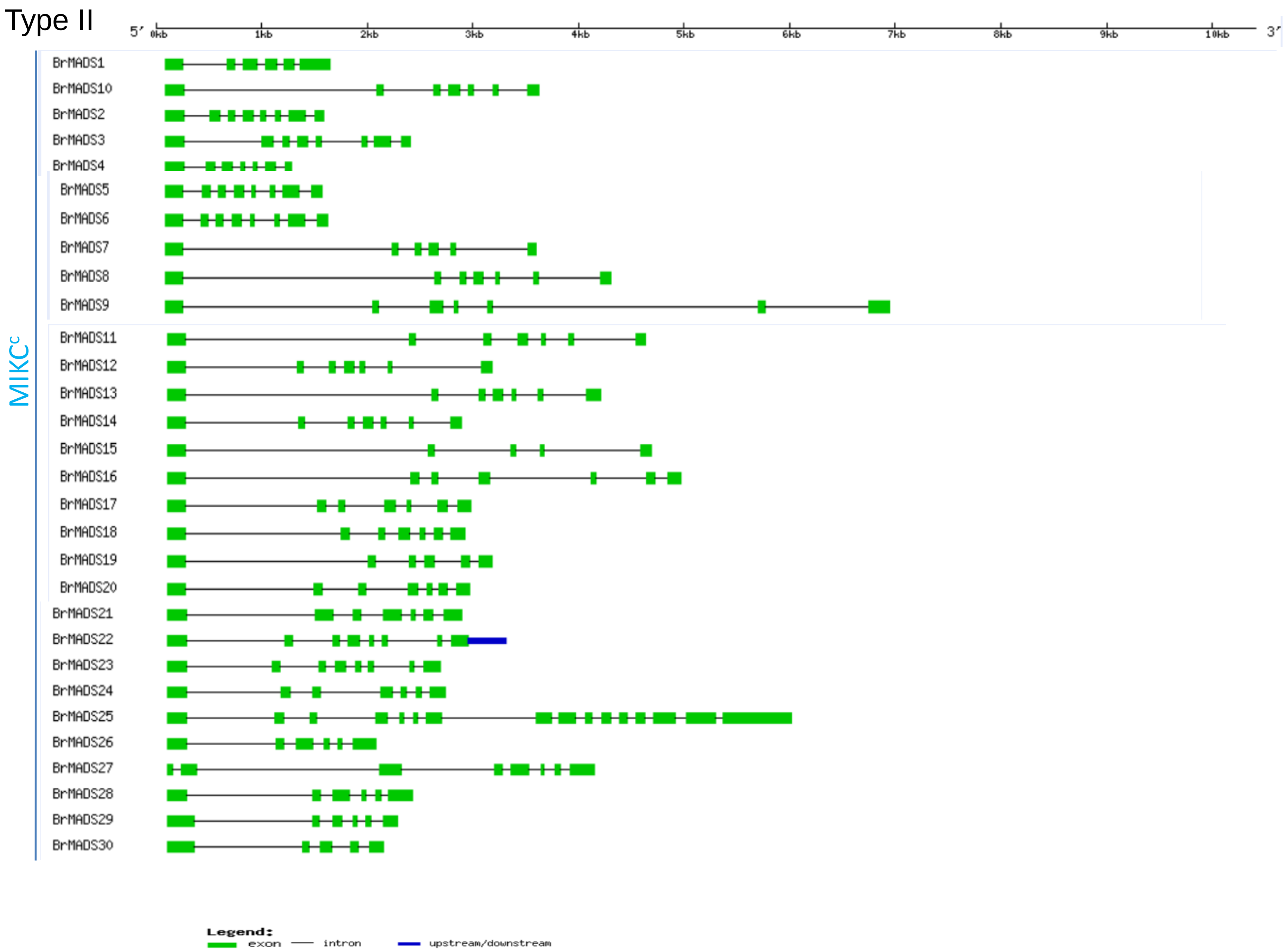

Type II
5′ 0kb 1kb 2kb 3kb 4kb 5kb 6kb 7kb 8kb 9kb 10kb 3′
MIKCc
BrMADS1
BrMADS10
BrMADS2
BrMADS3
BrMADS4
BrMADS5
BrMADS6
BrMADS7
BrMADS8
BrMADS9
BrMADS11
BrMADS12
BrMADS13
BrMADS14
BrMADS15
BrMADS16
BrMADS17
BrMADS18
BrMADS19
BrMADS20
BrMADS21
BrMADS22
BrMADS23
BrMADS24
BrMADS25
BrMADS26
BrMADS27
BrMADS28
BrMADS29
BrMADS30
Legend:
exon
intron
upstream/downstream

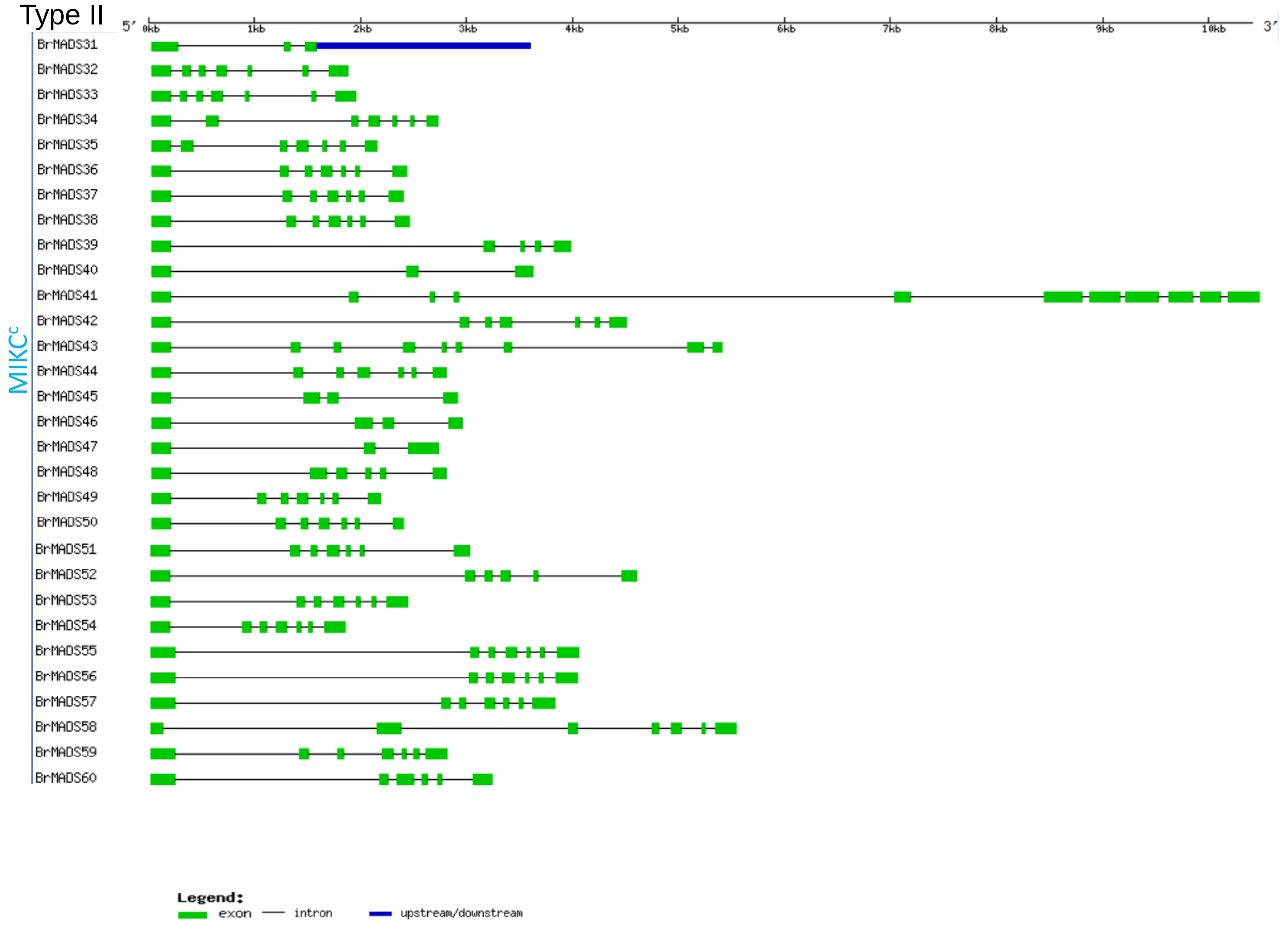
Type II
MIKC[c]
5′
3′
0kb
1kb
2kb
3kb
4kb
5kb
6kb
7kb
8kb
9kb
10kb
BrMADS31
BrMADS32
BrMADS33
BrMADS34
BrMADS35
BrMADS36
BrMADS37
BrMADS38
BrMADS39
BrMADS40
BrMADS41
BrMADS42
BrMADS43
BrMADS44
BrMADS45
BrMADS46
BrMADS47
BrMADS48
BrMADS49
BrMADS50
BrMADS51
BrMADS52
BrMADS53
BrMADS54
BrMADS55
BrMADS56
BrMADS57
BrMADS58
BrMADS59
BrMADS60
Legend:
exon
intron
upstream/downstream

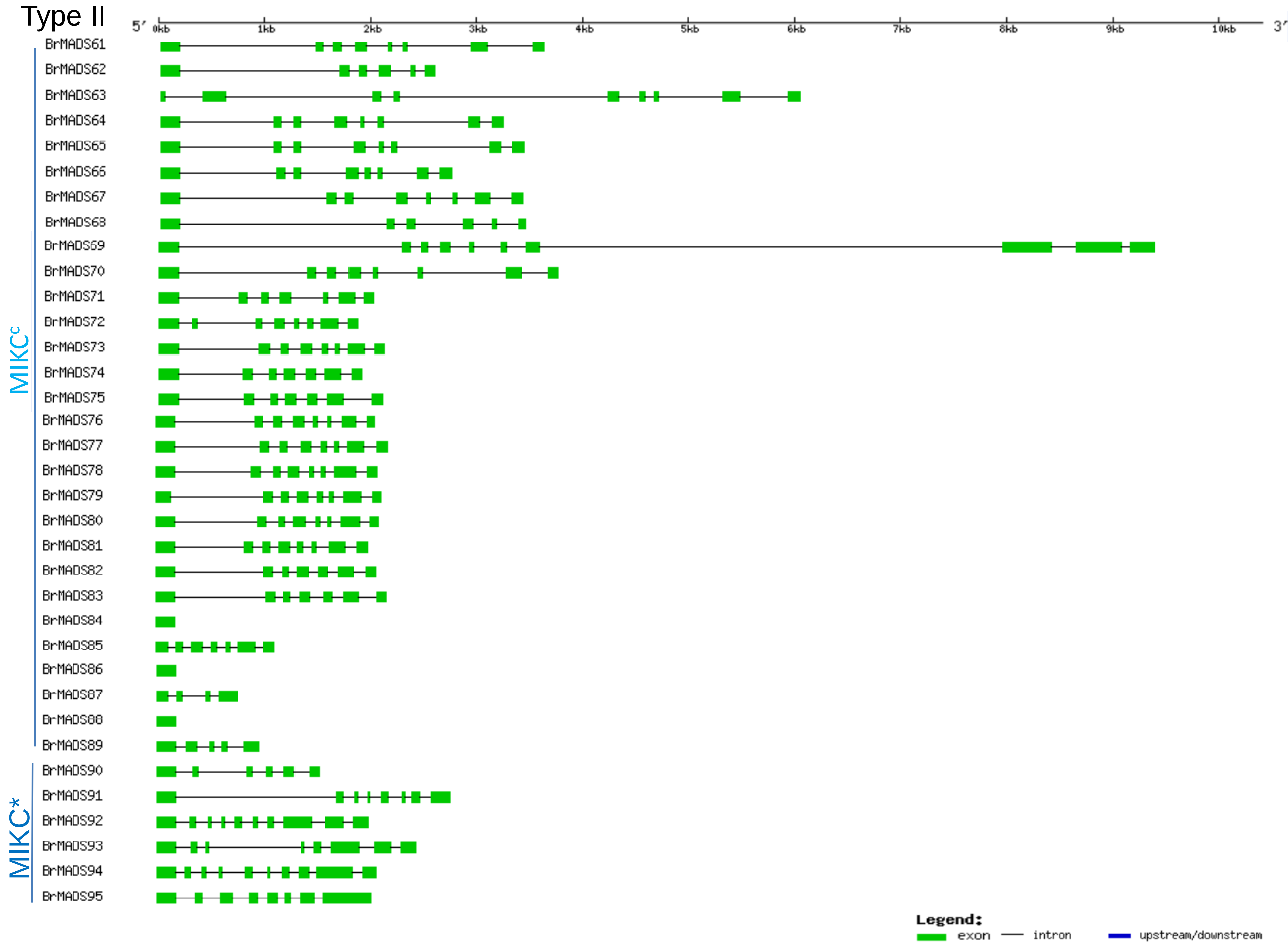

Type II
5′
0kb
1kb
2kb
3kb
4kb
5kb
6kb
7kb
8kb
9kb
10kb
3′
MIKCc
MIKC*
BrMADS61
BrMADS62
BrMADS63
BrMADS64
BrMADS65
BrMADS66
BrMADS67
BrMADS68
BrMADS69
BrMADS70
BrMADS71
BrMADS72
BrMADS73
BrMADS74
BrMADS75
BrMADS76
BrMADS77
BrMADS78
BrMADS79
BrMADS80
BrMADS81
BrMADS82
BrMADS83
BrMADS84
BrMADS85
BrMADS86
BrMADS87
BrMADS88
BrMADS89
BrMADS90
BrMADS91
BrMADS92
BrMADS93
BrMADS94
BrMADS95
Legend:
exon
intron
upstream/downstream

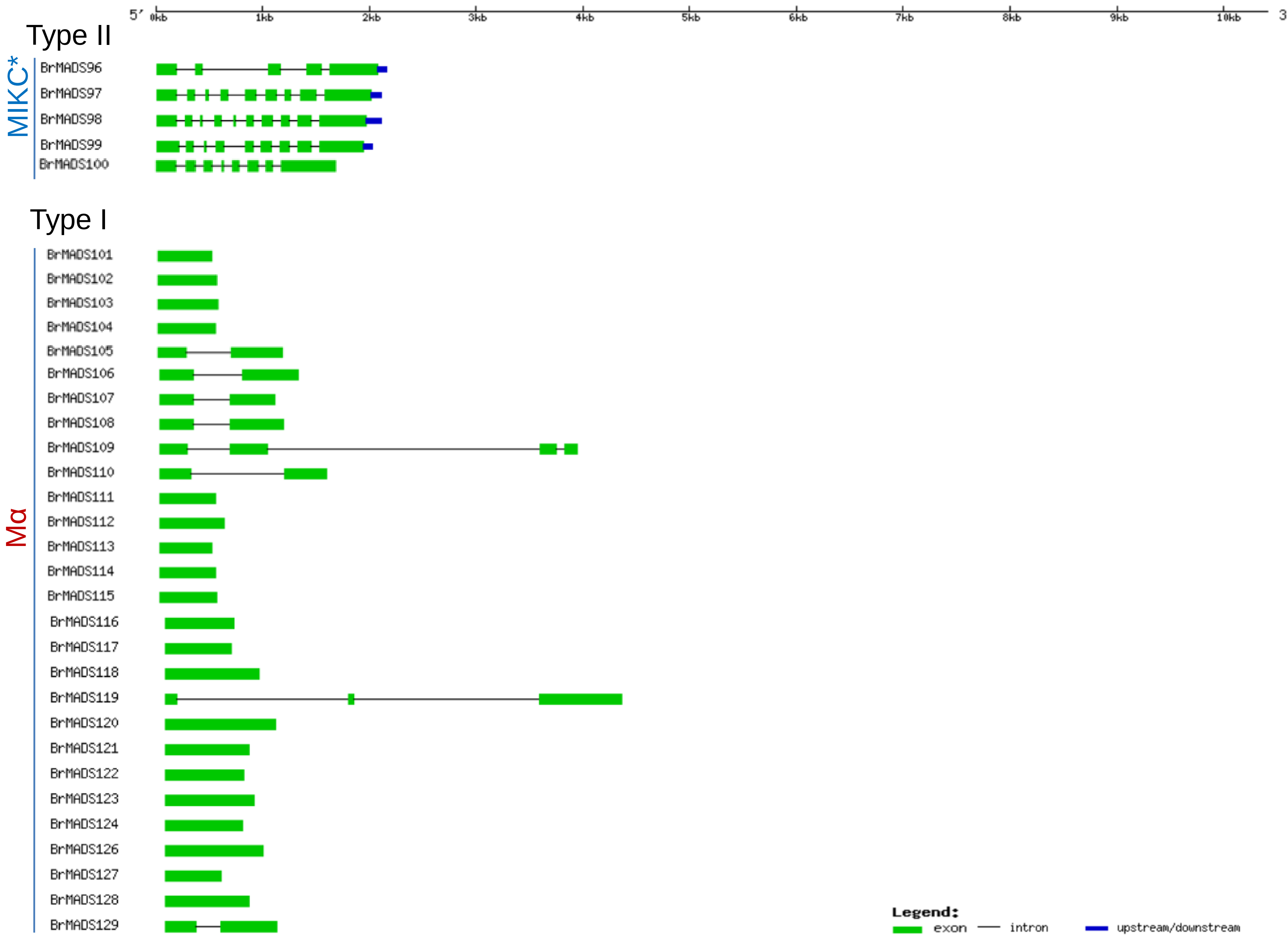

5′
0kb
1kb
2kb
3kb
4kb
5kb
6kb
7kb
8kb
9kb
10kb
3
Type II
MIKC*
BrMADS96
BrMADS97
BrMADS98
BrMADS99
BrMADS100
Type I
Mα
BrMADS101
BrMADS102
BrMADS103
BrMADS104
BrMADS105
BrMADS106
BrMADS107
BrMADS108
BrMADS109
BrMADS110
BrMADS111
BrMADS112
BrMADS113
BrMADS114
BrMADS115
BrMADS116
BrMADS117
BrMADS118
BrMADS119
BrMADS120
BrMADS121
BrMADS122
BrMADS123
BrMADS124
BrMADS126
BrMADS127
BrMADS128
BrMADS129
Legend:
exon
intron
upstream/downstream

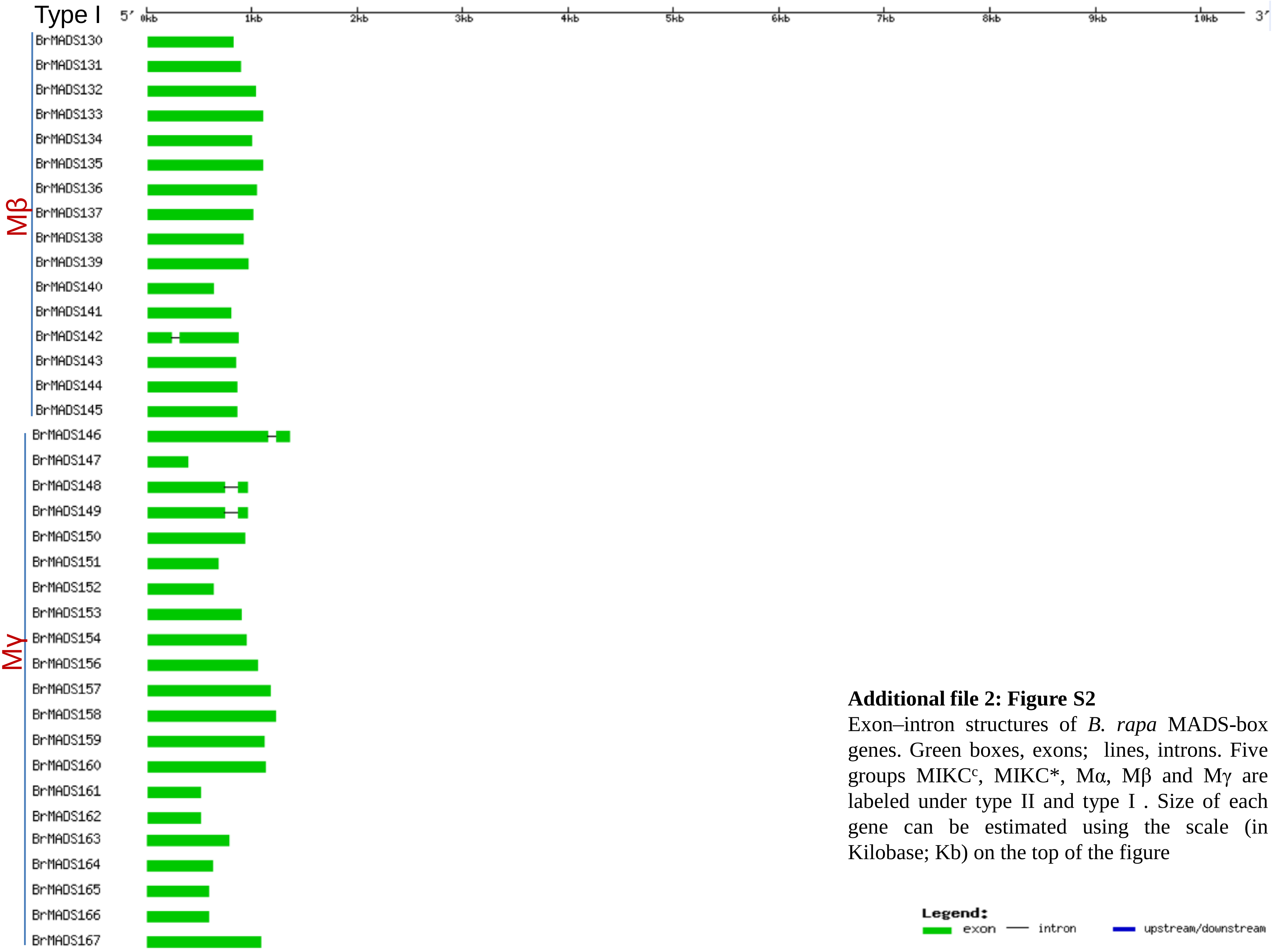


**Additional file 2: Figure S2**

Exon–intron structures of *B. rapa* MADS-box genes. Green boxes, exons; lines, introns. Five groups MIKC$^{c}$, MIKC*, Mα, Mβ and Mγ are labeled under type II and type I . Size of each gene can be estimated using the scale (in Kilobase; Kb) on the top of the figure

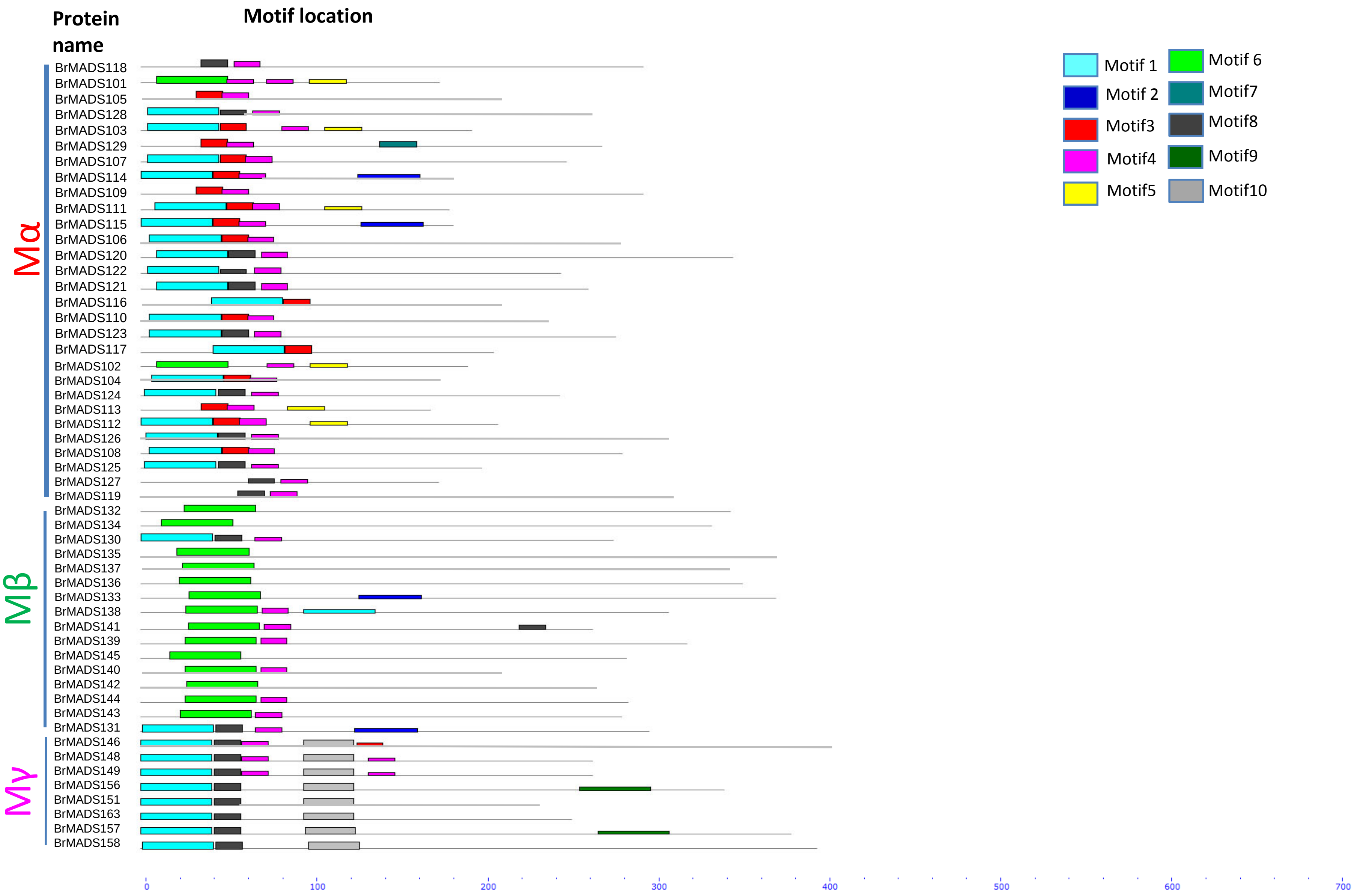

Protein name
Motif location
Motif 1
Motif 2
Motif3
Motif4
Motif5
Motif 6
Motif7
Motif8
Motif9
Motif10
Mα
Mβ
Mγ
BrMADS118
BrMADS101
BrMADS105
BrMADS128
BrMADS103
BrMADS129
BrMADS107
BrMADS114
BrMADS109
BrMADS111
BrMADS115
BrMADS106
BrMADS120
BrMADS122
BrMADS121
BrMADS116
BrMADS110
BrMADS123
BrMADS117
BrMADS102
BrMADS104
BrMADS124
BrMADS113
BrMADS112
BrMADS126
BrMADS108
BrMADS125
BrMADS127
BrMADS119
BrMADS132
BrMADS134
BrMADS130
BrMADS135
BrMADS137
BrMADS136
BrMADS133
BrMADS138
BrMADS141
BrMADS139
BrMADS145
BrMADS140
BrMADS142
BrMADS144
BrMADS143
BrMADS131
BrMADS146
BrMADS148
BrMADS149
BrMADS156
BrMADS151
BrMADS163
BrMADS157
BrMADS158
0
100
200
300
400
500
600
700

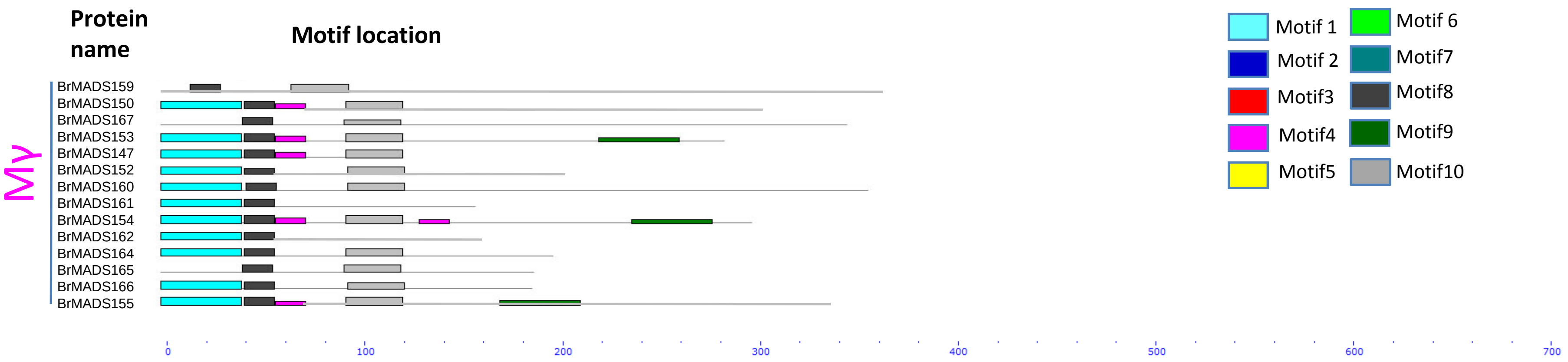


**Additional file 2: Figure S3**

**Distribution of Conserved motifs in *Brassica rapa* MADS-box type I proteins identified using MEME search tool**

Schematic representation of motifs identified in *B. rapa* MADS-box type I proteins using MEME motif search tool for each group (Mα, Mβ and Mγ) given separately. Different motifs are indicated by different colors, and the names of all members are shown on the left side of the figure. The order of the motifs corresponds to the position of the motifs in individual protein sequences

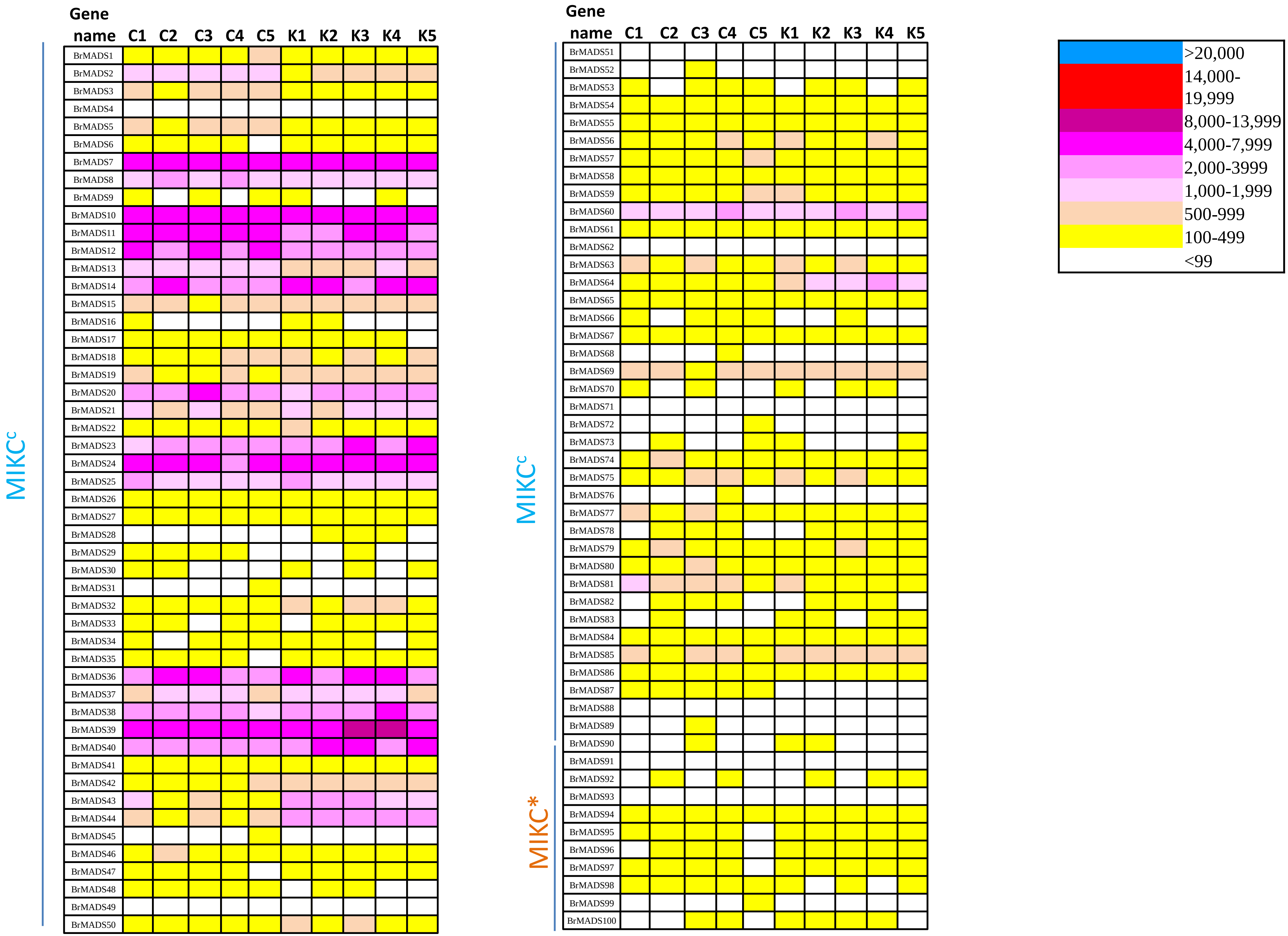

Gene name
C1 C2 C3 C4 C5 K1 K2 K3 K4 K5
BrMADS1
BrMADS2
BrMADS3
BrMADS4
BrMADS5
BrMADS6
BrMADS7
BrMADS8
BrMADS9
BrMADS10
BrMADS11
BrMADS12
BrMADS13
BrMADS14
BrMADS15
BrMADS16
BrMADS17
BrMADS18
BrMADS19
BrMADS20
BrMADS21
BrMADS22
BrMADS23
BrMADS24
BrMADS25
BrMADS26
BrMADS27
BrMADS28
BrMADS29
BrMADS30
BrMADS31
BrMADS32
BrMADS33
BrMADS34
BrMADS35
BrMADS36
BrMADS37
BrMADS38
BrMADS39
BrMADS40
BrMADS41
BrMADS42
BrMADS43
BrMADS44
BrMADS45
BrMADS46
BrMADS47
BrMADS48
BrMADS49
BrMADS50
MIKCc
Gene name
C1 C2 C3 C4 C5 K1 K2 K3 K4 K5
BrMADS51
BrMADS52
BrMADS53
BrMADS54
BrMADS55
BrMADS56
BrMADS57
BrMADS58
BrMADS59
BrMADS60
BrMADS61
BrMADS62
BrMADS63
BrMADS64
BrMADS65
BrMADS66
BrMADS67
BrMADS68
BrMADS69
BrMADS70
BrMADS71
BrMADS72
BrMADS73
BrMADS74
BrMADS75
BrMADS76
BrMADS77
BrMADS78
BrMADS79
BrMADS80
BrMADS81
BrMADS82
BrMADS83
BrMADS84
BrMADS85
BrMADS86
BrMADS87
BrMADS88
BrMADS89
BrMADS90
BrMADS91
BrMADS92
BrMADS93
BrMADS94
BrMADS95
BrMADS96
BrMADS97
BrMADS98
BrMADS99
BrMADS100
MIKCc
MIKC*
>20,000
14,000-19,999
8,000-13,999
4,000-7,999
2,000-3999
1,000-1,999
500-999
100-499
<99

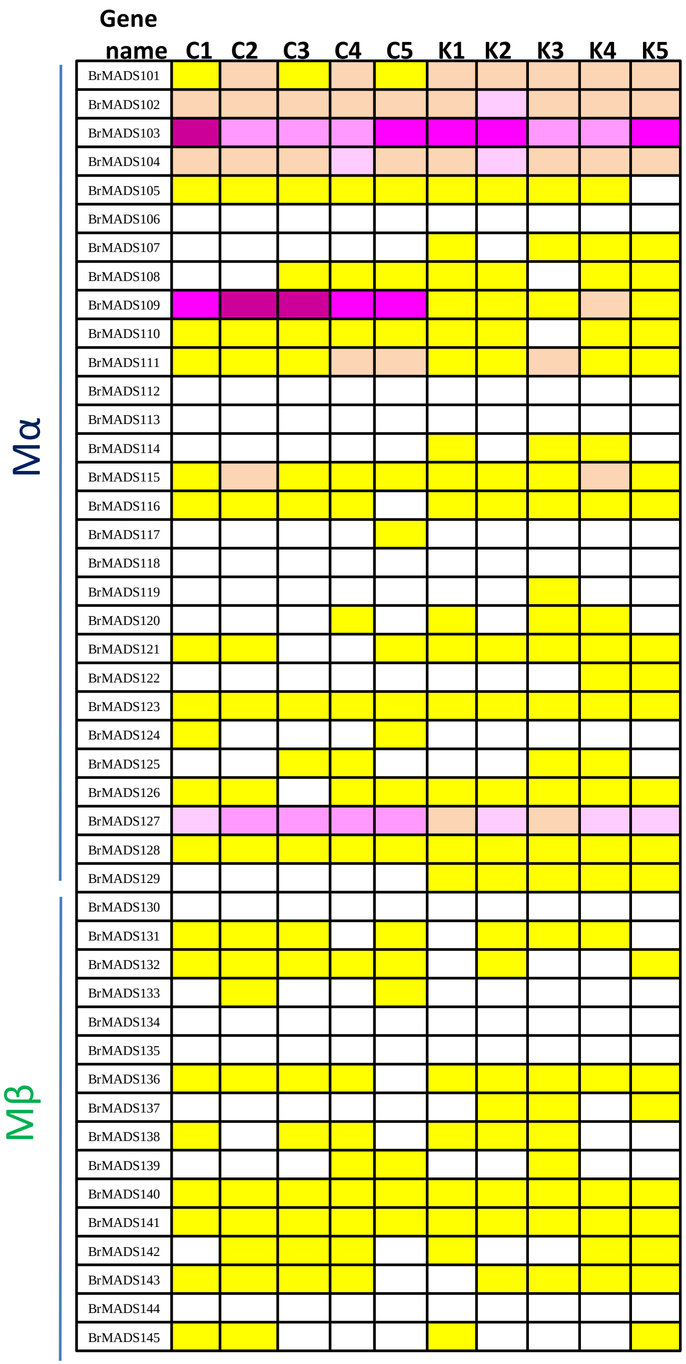


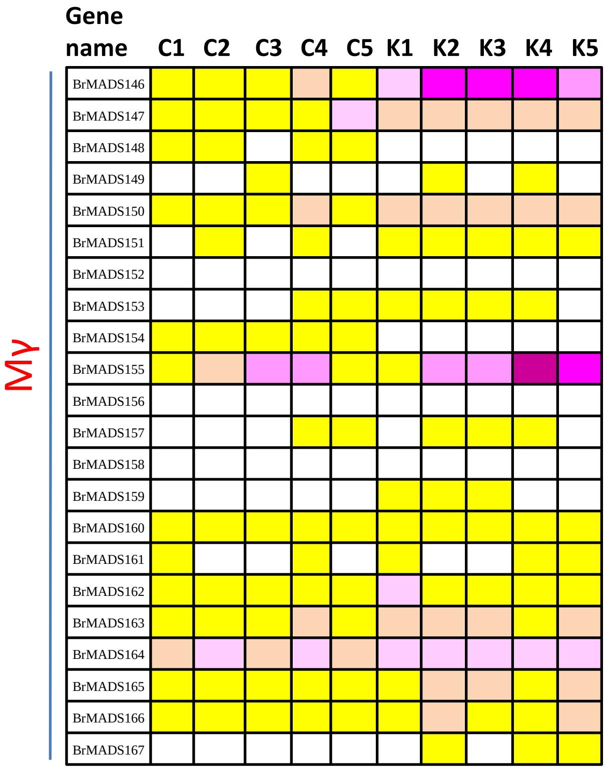


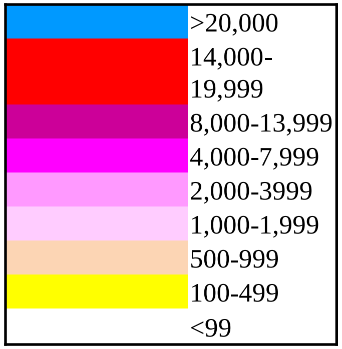


**Additional file 2: Figure S4**

Microarray expression analysis of MADS-box genes in *B. rapa* under different temperature treatment. Here C and K indicates Chiifu and Kenshin, were treated under five (5) temperatures as control (C1&K1), 4°c (C2&K2), 0°c (C3&K3), -2°c (C4&K4), and -4°c (C5&K5). Color bar at the top representing differential expression like purple representing medium level expression where pink to white showing low to no expression
